# A family of carboxypeptidases catalyzing α- and β-tubulin tail processing and deglutamylation

**DOI:** 10.1126/sciadv.adi7838

**Published:** 2023-09-13

**Authors:** Simon Nicot, Ghislain Gillard, Hathaichanok Impheng, Ewa Joachimiak, Serge Urbach, Kazufumi Mochizuki, Dorota Wloga, François Juge, Krzysztof Rogowski

**Affiliations:** ^1^ Tubulin Code team, Institute of Human Genetics, Université Montpellier, CNRS, Montpellier, France.; ^2^ Department of Physiology, Faculty of Medical science, Naresuan University, Phitsanulok 65000, Thailand.; ^3^ Laboratory of Cytoskeleton and Cilia Biology, Nencki Institute of Experimental Biology, Polish Academy of Sciences, 3 Pasteur Street, 02-093 Warsaw, Poland.; ^4^ Functional Proteomics Platform (FPP), IGF, Université Montpellier, CNRS, INSERM, Montpellier, France.; ^5^ Epigenetic Chromatin Regulation team, Institute of Human Genetics, Université Montpellier, CNRS, Montpellier, France.

## Abstract

Tubulin posttranslational modifications represent an important mechanism involved in the regulation of microtubule functions. The most widespread among them are detyrosination, α∆2-tubulin, and polyglutamylation. Here, we describe a family of tubulin-modifying enzymes composed of two closely related proteins, KIAA0895L and KIAA0895, which have tubulin metallocarboxypeptidase activity and thus were termed TMCP1 and TMCP2, respectively. We show that TMCP1 (also known as MATCAP) acts as α-tubulin detyrosinase that also catalyzes α∆2-tubulin. In contrast, TMCP2 preferentially modifies βI-tubulin by removing three amino acids from its C terminus, generating previously unknown βI∆3 modification. We show that βI∆3-tubulin is mostly found on centrioles and mitotic spindles and in cilia. Moreover, we demonstrate that TMCPs also remove posttranslational polyglutamylation and thus act as tubulin deglutamylases. Together, our study describes the identification and comprehensive biochemical analysis of a previously unknown type of tubulin-modifying enzymes involved in the processing of α- and β-tubulin C-terminal tails and deglutamylation.

## Teaser

Discovery of an enzyme family processing tubulin C-terminal tails reveals a previously unknown β-tubulin modification.

## INTRODUCTION

Microtubules (MTs) are essential cytoskeletal elements composed of α/β-tubulin heterodimers. They are involved in many important functions such as intracellular transport, cell division, cell motility, and morphogenesis. Functional adaptation of MTs is often achieved through posttranslational modifications. Most tubulin modifications occur at the C-terminal tails, which are present on the MT surface where they provide the key interaction sites for MT-associated proteins (MAPs) and molecular motors. The most prevalent modifications are detyrosination, which consists in the removal of the very C-terminal tyrosine from α-tubulin resulting in the generation of the so-called α∆1-tubulin, and polyglutamylation, which involves the addition of glutamate side chains to both tubulins.

Detyrosination was the first tubulin modification to be discovered (*1*) and since then shown to regulate various MAPs and molecular motors. It acts as a positive regulator of kinesin-1 (Kif5B) (*2*) and kinesin-7 (CENP-E) (*3*) while it inhibits the activity of kinesin-13 (*4*) and the binding of CAP-Gly domain–containing proteins such as cytoplasmic linker protein of 170 kDa (CLIP170) and p150^Glued^, a component of the dynein-dynactin complex (*5*). Tubulin detyrosination plays an important regulatory role in cell division (*3*), cell migration (*6*), neuronal physiology (*2*, *7*), and cardiac mechanotransduction (*8*). Hence, this modification has been implicated in various disorders including cancer (*9*, *10*), cardiomyopathies (*11*), heart failure (*12*), and neurodegeneration (*13*).

Recently, the two members of the vasohibin family (VASH1 and VASH2) in complex with their essential cofactor small vasohibin-binding protein (SVBP) were shown to catalyze the removal of the tyrosine (*14*, *15*). The identification of VASHs revealed the existence of at least one additional tubulin detyrosinase (*14*). In contrast, the reverse reaction is catalyzed by a single enzyme called tubulin tyrosine ligase (TTL) (*16*).

**Fig. 1.**
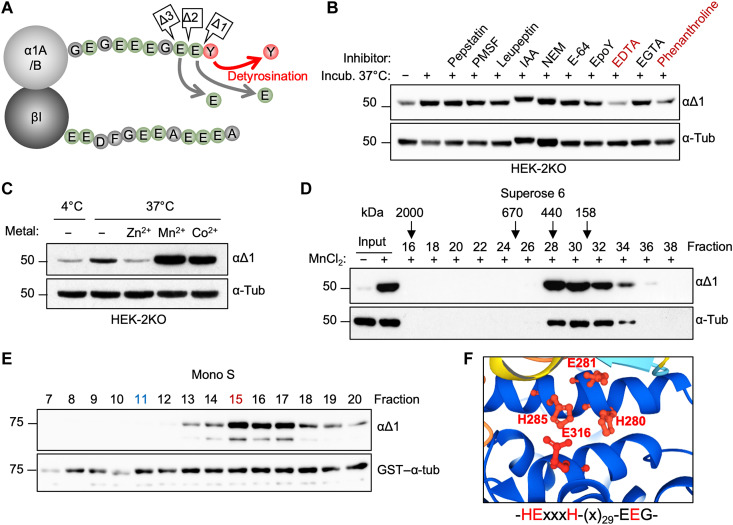
Purification and identification of α-tubulin detyrosinase. (**A**) Schematic representation of the known α-tubulin C-terminal cleavages, which generate detyrosinated (αΔ1), αΔ2-, and αΔ3-tubulins. (**B**) Immunoblots of an in vitro assay testing the effect of inhibitors specific to different types of proteases on the endogenous detyrosinase activity from HEK-2KO cells. PMSF, phenylmethylsulfonyl fluoride; IAA, iodoacetamide; NEM, *N*-ethylmaleimide. (**C**) Immunoblot of an in vitro assay testing the effect of different metal ions on the endogenous detyrosinase activity from HEK-2KO cells. (**D**) Immunoblots of detyrosinase activity in different fractions following gel filtration assayed on endogenous tubulin. Fraction numbers and molecular mass standards used to calibrate the column are indicated on the top. (**E**) Immunoblots of detyrosinase activity in different fractions after cation-exchange chromatography (Mono S) of the enriched detyrosinase activity after the first step of purification. The activity was measured against recombinant GST–α-tubulin, which was added to each fraction. Fractions 11 (blue) and 15 (red) were subjected to mass spectrometry analysis as a negative and positive fraction, respectively. (**F**) AlphaFold-based prediction of the putative active site of KIAA0895L.

Detyrosinated tubulin can be further processed by sequential removal of one or two glutamates, which is catalyzed by members of the cytosolic carboxypeptidase (CCP) family resulting in the formation of α∆2- or α∆3-tubulin (Fig. 1A) (*17*, *18*). Apart from deglutamylation of the α-tubulin primary chain, CCPs also remove posttranslational polyglutamylation, which is added by members of the tubulin tyrosine ligase like (TTLL) family (*19*). The human genome encodes nine glutamylases, among which six have autonomous activity, with TTLL4, TTLL5, and TTLL7 being involved mostly in the initiation while TTLL6, TTLL11 and TTLL13 are highly efficient at catalyzing the elongation of the glutamate side chains (*19*). The remaining three glutamylases—TTLL1, TTLL2 and TTLL9—are inactive on their own, and they are most likely a part of larger complexes requiring other subunits for their activity as exemplified by TTLL1, which forms a complex with four additional proteins (*20*).

The removal of glutamyl side chains is also a two-step process. Among six CCP family members, most (CCP1 to CCP4 and CCP6) are involved in shortening the glutamate chain, while CCP5 specifically removes the branching point glutamates (*17*, *21*).

At the molecular level, polyglutamylation has been shown to regulate MT stability either by affecting the binding of classical MAPs such as MAP1, MAP2 (*22*), and tau (*23*) or by controlling the activity of MT severing enzymes including spastin and katanin (*24*–*26*). Moreover, this modification also regulates the activity of certain kinesins as well as cytoplasmic and ciliary dyneins (*27*). Dysregulation of polyglutamylation was found to be associated with several pathologies including neurodegeneration (*17*, *28*, *29*), cancer progression (*30*), and various ciliopathies (*31*) underlying the importance of maintaining proper levels of this modification.

Here, we describe the discovery of a family of tubulin-modifying enzymes composed of two members, TMCP1 and TMCP2, which catalyze the removal of amino acids from α- and β-tubulin C-terminal tails. TMCP1, which corresponds to the recently described enzyme called MT-associated tyrosine carboxypeptidase (MATCAP) (*32*), is specific to α-tubulin and acts as a highly efficient detyrosinase. However, it also generates αΔ2-tubulin through sequential removal of tyrosine and glutamate. In contrast, TMCP2 catalyzes primarily the modification of βI-tubulin from which it sequentially removes three residues generating a previously unknown βIΔ3 modification. Using newly developed modification-specific antibodies, we show that βIΔ3-tubulin is present on centrioles, mitotic spindles, and cilia in various cell lines of different origins suggesting that it may play a role in cell division and ciliogenesis. Moreover, we demonstrate that both TMCPs also remove posttranslational polyglutamylation and thus act as tubulin deglutamylases. In agreement, we further show that TMCP homolog from *Tetrahymena*, an evolutionary distant organism, is also a tubulin deglutamylase specifically involved in the regulation of polyglutamylation levels at the basal bodies.

## RESULTS

### Identification and characterization of TMCP1

To identify the missing detyrosinase(s), we have developed a biochemical approach using extracts from human embryonic kidney (HEK) 293 cells knockout for both *VASHs* [HEK-2KO; (*14*)]. We set up an in vitro assay, which measured detyrosination activity toward endogenous α-tubulin, and used it to test various inhibitors specific to different classes of proteases. Because detyrosinase activity was inhibited exclusively by ion chelating agents such as EDTA and phenanthroline (Fig. 1B), it suggests that the unknown enzyme is a metalloprotease. To confirm this hypothesis, we performed an in vitro assay in the presence of metals that are commonly used by metalloproteases such as zinc, manganese, and cobalt. While zinc had an inhibitory effect, the addition of manganese or cobalt strongly increased detyrosinase activity (Fig. 1C). These data confirm that the unidentified enzyme is indeed a metalloprotease.

To enrich for the enzymatic activity present in the HEK-2KO cells, we developed a two-step purification procedure, which consisted of a gel filtration and cation-exchange chromatography. Following gel filtration, we assayed detyrosinase activity using the endogenous tubulin as a substrate. The highest detyrosinase activity was detected in fractions 28 to 32 (Fig. 1D), which were pooled together and subjected to cation-exchange chromatography. Because the negatively charged tubulins do not bind to cation-exchange resin, we supplemented the reactions with bacterially produced glutathione *S*-transferase (GST)–α-tubulin to monitor the activity. The highest activity was present in fraction 15 (Fig. 1E). Thus, we analyzed the protein composition of this fraction by mass spectrometry and compared it to fraction 11, which had no activity. Consequently, we established a list of proteins that were specifically enriched in the active fraction (table S1).

To identify the potential candidates for being α-tubulin detyrosinase, we focused on metal binding motifs characteristic for the three most common metallopeptidase tribes. This included the “HxxE” motif typical for αβα-Exopeptidases and “HEx_2–3_H” or “HxxEH” found in zincins and inverzincins, respectively (*33*). Because the unknown detyrosinase was not inhibited by various inhibitors specific to different families belonging to the αβα-Exopeptidases tribe (fig. S1A), we searched for candidates containing one of the two remaining motifs. Among the top hits (table S1), we identified KIAA0895L, a protein of unknown function that was present exclusively in the active fraction. It contains a “HExxxH” motif characteristic of M49 metalloprotease family [MEROPS database; (*34*)]. Furthermore, the three-dimensional (3D) structure of KIAA0895L predicted by AlphaFold (*35*) showed that the protein has a folded globular domain in which the HExxxH motif is positioned in close proximity to a putative third metal-binding residue located in a characteristic E**E**G motif [Fig. 1F and fig. S1B (*36*)]. Together, this suggests that KIAA0895L might have a metalloprotease activity.

**Fig. 2.**
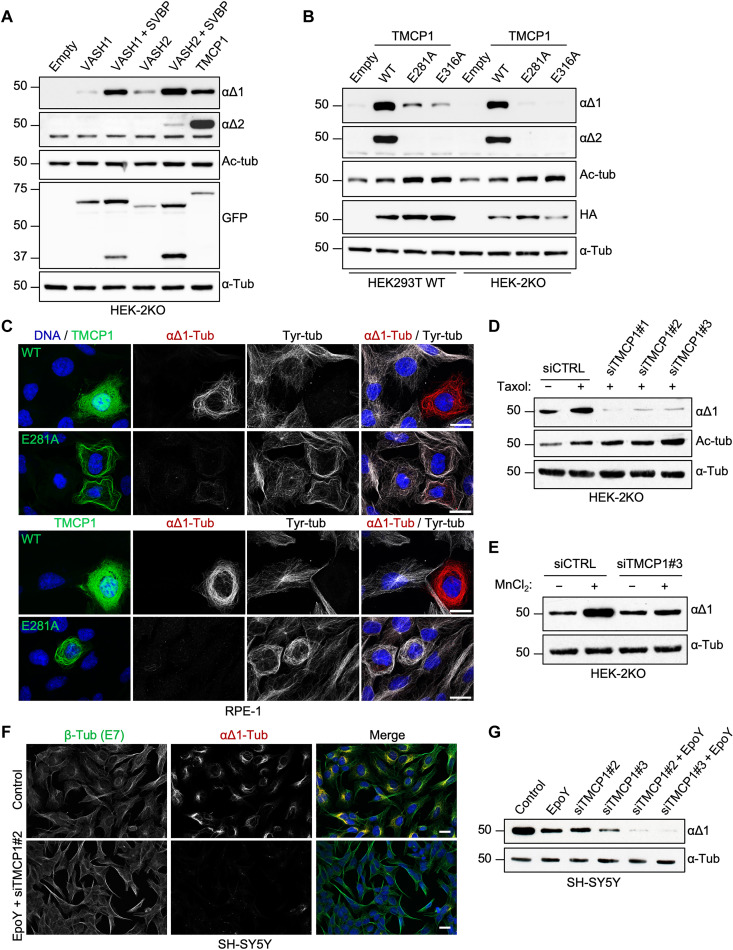
TMCP1 catalyzes αΔ1- and αΔ2-tubulin. (**A**) Immunoblots of protein extracts from HEK-2KO cells overexpressing VASH1 and VASH2, either alone or in combination with SVBP, and TMCP1. (**B**) Immunoblots of protein extracts from HEK293T or HEK-2KO cells expressing either the WT or enzymatically inactive mutants (E281A and E316A) of HA-TMCP1. (**C**) Immunofluorescence analysis of RPE1 cells expressing either active or inactive (E281A) GFP-TMCP1. Note the apparent MT bundling specific to cells expressing inactive GFP-TMCP1. Scale bars, 20 μm. (**D**) Immunoblots of protein extracts from HEK-2KO cells treated with paclitaxel following knock down of *TMCP1* using three different siRNAs. (**E**) Immunoblots of an in vitro assay measuring tubulin detyrosination activity in control and TMCP1-depleted HEK-2KO cells. (**F**) Immunofluorescence analysis of SH-SY5Y cell knockdown for *TMCP1* and simultaneously treated with the VASH inhibitor EpoY. (**G**) Immunoblots of protein extracts from SH-SY5Y cells treated with the EpoY inhibitor, following the knockdown of *TMCP1* or after combination of the two treatments.

To test whether KIAA0895L is involved in tubulin detyrosination, we expressed it in HEK-2KO cells and compared its activity either with VASH1 and VASH2 alone or in combination with their essential activator, SVBP. The expression of KIAA0895L increased the levels of α∆1-tubulin to similar extent as VASH1 or VASH2 coexpressed with SVBP (Fig. 2A). Thus, we have named KIAA0895L a tubulin metallocarboxypeptidase 1 (TMCP1). To assess whether TMCP1 is strictly a detyrosinase, we tested whether it can also generate α∆2 modification. Notably, the expression of TMCP1 resulted in a strong increase of α∆2-tubulin levels (Fig. 2, A and C), suggesting that it has a dual activity and can generate both modifications. Furthermore, single-point mutations of the predicted catalytic or metal-binding glutamates (E281A and E316A) strongly reduced the activity of TMCP1 in HEK293 cells (Fig. 2B). The remaining α∆1 modification was likely generated by VASHs in response to MT stabilization that, as demonstrated by accumulation of tubulin acetylation (Fig. 2B) and MT bundling (Fig. 2C and fig. S1C), was caused exclusively by TMCP1 mutants. In agreement, when corresponding proteins were expressed in HEK-2KO cells, the residual α∆1 signal was abolished (Fig. 2B). These data provide further evidence that TMCP1 is indeed involved in modifying α-tubulin.

Next, to test whether the endogenous TMCP1 contributes to detyrosination, we performed knockdown experiments in HEK-2KO cells using three different small interfering RNAs (siRNAs). We observed almost complete depletion of α∆1-tubulin signal (Fig. 2D and fig. S1D), even in the presence of paclitaxel, which is known to promote detyrosination due to its MT-stabilizing properties (*37*). In addition, the extracts from HEK-2KO cells depleted for TMCP1 showed a strong reduction in detyrosinase activity (Fig. 2E). Thus, TMCP1 is responsible for VASH-independent detyrosinase activity present in HEK293 cells.

Last, to assess the individual contribution of the two types of detyrosinating enzymes to overall levels of αΔ1-tubulin, we used SH-SY5Y cell line, which is characterized by high levels of this modification. We inhibited the activity of VASHs using EpoY inhibitor, while the expression of *TMCP1* was knockdown with two different siRNAs. When applied individually, both treatments partially reduced the level of detyrosination, while their combination led to a complete elimination of αΔ1 modification (Fig. 2, F and G). This shows that both VASH- and TMCP1-dependent activities are involved in the generation of αΔ1-tubulin and that they are most likely the only detyrosinating enzymes present in SH-SY5Y cells.

**Fig. 3.**
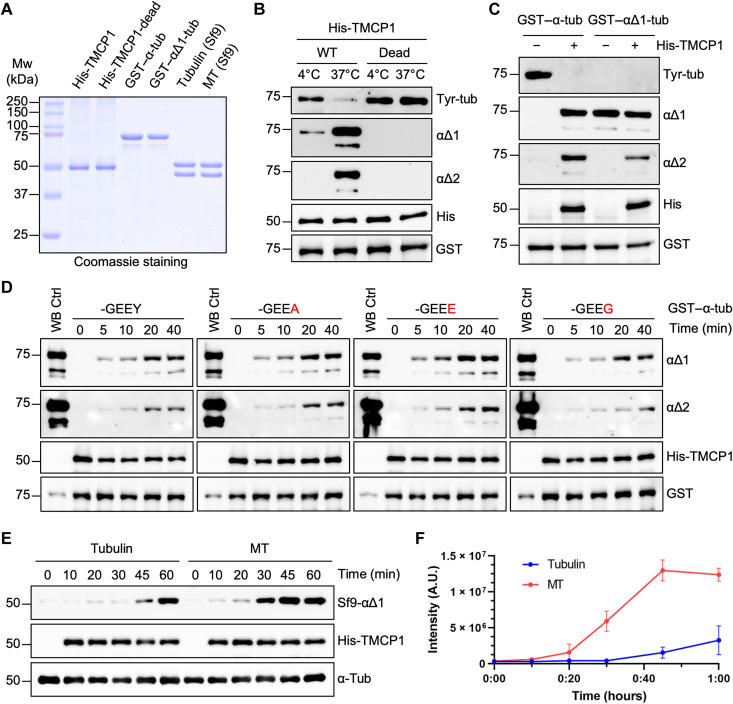
Enzymatic mechanism and substrate specificity of TMCP1. (**A**) Coomassie staining of purified proteins used for in vitro studies of TMCP1. His-TMCP1, His-TMCP1-E281A, GST–α-tubulin, and GST–αΔ1-tubulin were expressed in BL21 bacteria, whereas tubulin and MT were purified and assembled from insect (Sf9) cells. Mw, molecular weight. (**B**) Immunoblots of an in vitro assay using recombinant His-TMCP1 or its catalytically inactive version (E281A) and bacterially produced GST–α-tubulin. (**C**) Immunoblots of an in vitro assay involving bacterially produced GST–α-tubulin or GST–αΔ1-tubulin treated with recombinant His-TMCP1. (**D**) Immunoblots of an in vitro time-course assay using recombinant His-TMCP1 and either the WT GST–α-tubulin or its mutated versions in which the C-terminal tyrosine has been replaced by alanine, glutamate, or glycine. WB, Western blot. (**E**) Immunoblots of an in vitro assay measuring time-dependent activity of recombinant His-TMCP1 toward Sf9-derived tubulin dimers or MTs. (**F**) Graphical representation of His-TMCP1 activity toward Sf9-derived tubulin or MTs. Immunoblot signals were quantified for each time point (mean ± SD; *n* = 3 independent experiments). A.U., arbitrary units.

To confirm that TMCP1 directly modifies α-tubulin, we performed an in vitro assay using either wild-type (WT) or catalytically dead (E281A) recombinant enzyme and bacterially expressed GST–α-tubulin as a substrate (Fig. 3A). Active enzyme generated α∆1- and α∆2-tubulin signal with concomitant reduction in the tyrosination levels, whereas E281A mutant was inactive (Fig. 3B). These data demonstrate that TMCP1 catalyzes not only α∆1- but also α∆2-tubulin.

To test whether the generation of α∆2-tubulin by TMCP1 could result from sequential removal of the C-terminal tyrosine followed by penultimate glutamate, we compared the activity of TMCP1 using either the tyrosinated or α∆1-tubulin. We found that TMCP1 was highly efficient at generating α∆1 modification from tyrosinated tubulin and that it produced α∆2-tubulin from both substrates (Fig. 3C). These results support sequential formation of α∆2 modification by TMCP1.

Next, we evaluated the specificity of TMCP1 toward recombinant α-tubulin substrates carrying either tyrosine, alanine, glutamate or glycine at their very C terminus. Time course experiments showed that TMCP1 modifies all four substrates with similar efficiency (Fig. 3D). Because recombinant substrates might have not been properly folded, we confirmed our results using tubulin dimers carrying the same C-terminal endings purified from HEK293 cells expressing α-tubulins with internal His-tag (*38*). As shown by immunofluorescence analysis, the addition of the His-tag did not affect the incorporation of the tubulin into the MTs (fig. S2A), demonstrating that it is likely to be properly folded. As observed for recombinant substrates, tubulin dimers were modified to similar extent regardless of the ending (fig. S2B).

Subsequently, we compared the specificity of TMCP1 and VSH2/SVBP by coexpressing either the WT or catalytically inactive enzymes with green fluorescent protein (GFP)–α-tubulin variants carrying various C-terminal tails in HEK293 cells. TMCP1 generated α∆1-tubulin from substrates ending with tyrosine, phenylalanine, and alanine (fig. S2C). In contrast, the substrates in which the penultimate glutamate has been replaced by aspartate or glutamine (-GEDY or -GEQY) were not modified. Similar results were obtained when the more distal glycine residue was substituted by a glutamate (-GEEY versus -EEEY; fig. S2C). In comparison, VASH2/SVBP modified most variants except the one carrying C-terminal alanine or penultimate aspartate (fig. S2D), highlighting differences in specificity between the two types of enzymes.

Next, given that detyrosination preferentially accumulates on MTs, we compared the activity of TMCP1 toward tubulin and MTs in vitro. We used tubulin purified from insect cells (Fig. 3A), which is fully tyrosinated (*39*). We found that the enzyme modified MTs more efficiently than tubulin dimers (Fig. 3, E and F), a preference shared with VASHs (*15*).

Last, we evaluated metal dependence of recombinant TMCP1 by performing metal replacement experiments involving zinc, manganese, and cobalt. Comparison of the inhibitory properties of EDTA and EGTA confirmed that the former is a much more potent inhibitor of TMPC1 activity (fig. S2E). Following metal stripping with EDTA, we found that manganese and cobalt activated TMCP1 in a dose-dependent manner, whereas the addition of zinc was mostly inhibitory (fig. S2F). These results are consistent with the assays involving the endogenous enzyme (Fig. 1C).

### Identification and characterization of TMCP2

**Fig. 4.**
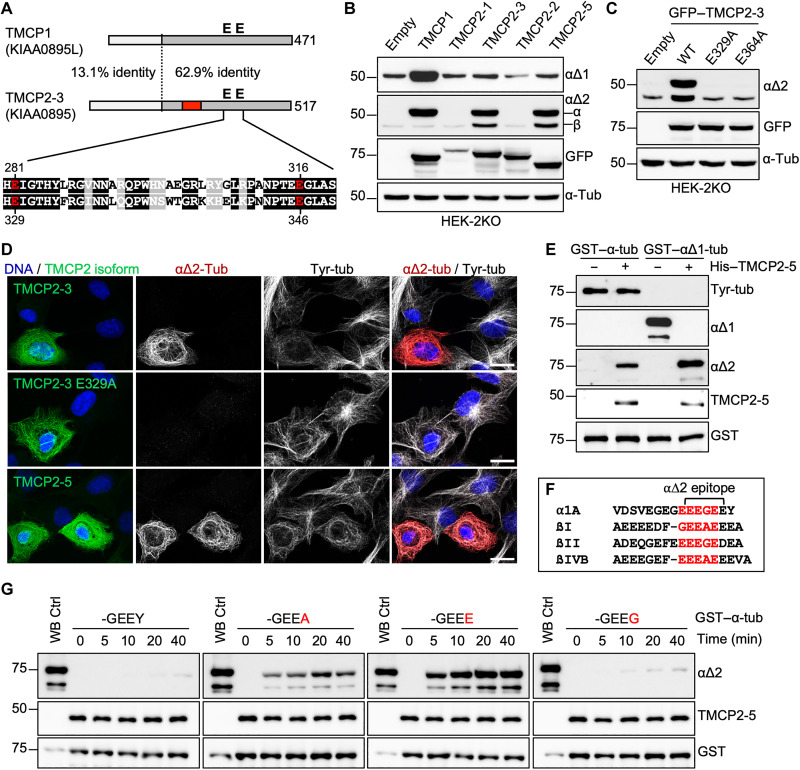
Characterization of TMCP2, a paralog of TMCP1. (**A**) Schematic alignment of TMCP1 and TMCP2 (isoform 3) shows a high degree of conservation in the C-terminal region that contains the active site. The region encoded by the alternative exon is indicated in red. Sequence alignment of the active site region with highlighted essential glutamates is presented below. (**B**) Immunoblots of protein extracts from HEK-2KO cells expressing TMCP1 and several isoforms of TMCP2. (**C**) Immunoblots of protein extracts from HEK-2KO cells expressing TMCP2-3 or its enzymatically inactive versions (E329A and E364A). (**D**) Immunofluorescence analysis of RPE1 cells expressing TMCP2-3 or its enzymatically inactive version (E329A) and TMCP2-5. Scale bars, 20 μm. (**E**) Immunoblots of an in vitro assay involving recombinant His-TMCP2-5 and bacterially produced GST–α-tubulin or GST–αΔ1-tubulin. (**F**) Schematic representation of the potential epitopes (highlighted in red) recognized by αΔ2 antibody present in the C terminus of α-, βI-, βII-, and βIV-tubulin. (**G**) Immunoblots of an in vitro time-course assay using recombinant His-TMCP2-5 in the presence of either the WT GST–α-tubulin or its mutated versions in which the C-terminal tyrosine has been replaced by alanine, glutamate, or glycine.

Blast searches of human genome using the sequence of KIAA0895L revealed the existence of a close homolog, KIAA0895 (Fig. 4A). Conservation of the active site residues in KIAA0895 (Fig. 4A) suggests that it is likely to be active, and thus, we termed it TMCP2. According to the National Center for Biotechnology Information database, *TMCP2* locus is predicted to generate eight different protein isoforms through the use of multiple promoters and alternative splicing (fig. S3A). We overexpressed several isoforms in HEK-2KO cells and assessed their ability to increase the levels of α∆1- or α∆2-tubulin. While the overexpression of TMCP2-1 and TMCP2-2 did not affect the levels of α∆1- or α∆2-tubulin, the expression of TMCP2-3 and TMCP2-5 resulted in an increase of α∆2- but not α∆1-tubulin (Fig. 4, B and D). Furthermore, mutations of the active-site glutamates in TMCP2-3 (E329A or E346A) abolished the activity (Fig. 4, C and D). The main difference between the active and inactive TMCP2 isoforms is the presence or absence of the alternative exon (fig. S3A), which appears to be required for the activity. Upon overexpression, TMCP2-3 and TMCP2-5 were found to be associated with MTs (fig. S3B). In contrast, TMCP2-1 showed no MT association and was found mostly in the nucleus (fig. S3B), which was consistent with the presence of the putative nuclear localization signal at its N terminus (fig. S3A). TMCP2-2, which differs from TMCP2-3 only by the lack of the alternative exon (fig. S4, A and B), was mostly found in the cytosol but not colocalizing with MTs (fig. S3B). Together, our data suggest that the alternative exon is involved in mediating the interaction of TMCP2 with MTs, which could explain its essential role in the activity.

Because TMCP2 appeared to directly generate α∆2-tubulin (Fig. 4B), we tested whether the formation of this modification might be the result of sequential removal of single amino acids as was the case for TMCP1 (Fig. 3C). Thus, we set up an in vitro assay to compare the activity of recombinant TMCP2 (fig. S5A) toward either tyrosinated or α∆1-tubulin (Fig. 3A). The enzyme generated α∆2-tubulin from both variants; however, the processing of α∆1-tubulin was more efficient (Fig. 4E). Thus, the generation of α∆2 is likely to be sequential, with TMCP2 removing glutamates more efficiently than tyrosine. This conclusion was further supported by the time-course experiments with recombinant α-tubulins carrying either tyrosine, alanine, glutamate, or glycine at its C terminus. We found that TMCP2 was the most active on the substrate ending with glutamate (Fig. 4G). A slightly lower activity was observed on tubulin ending with alanine, while the substrates ending with tyrosine or glycine were poorly modified (Fig. 4G). Our results were further confirmed on tubulin dimers ending with the same amino acids purified from HEK293 cells using engineered α-tubulins with internal His-tag (fig. S5B). Thus, in contrast to TMCP1, which removed all four amino acids with similar efficiency, TMCP2 shows a strong preference for glutamates and alanine over glycine or tyrosine. The more efficient removal of glutamates as compared to tyrosine could explain the generation of α∆2-tubulin by TMCP2 without an increase in detyrosination.

Subsequently, we evaluated the specificity of TMCP2, by coexpressing either the WT or enzymatically dead (E329A) enzyme with His–α-tubulins carrying various C-terminal tails in HEK293 cells. We found that TMCP2 generated α∆2 modification from substrates ending with tyrosine, phenylalanine, and alanine (fig. S5C). In contrast, the substrates carrying mutations of the more proximal amino acids (-GEDY, -GEQY, and -EEEY) were not modified (fig. S5C). Thus, the activity of both TMCPs showed similar sensitivity to mutations of the penultimate glutamate or the more proximal glycine residue.

Next, we tested the metal requirements of TMCP2, which, similarly to TMCP1, was also preferentially inhibited by EDTA as compared to EGTA (fig. S5D). Metal replacement experiments demonstrated that all three metals including zinc, manganese, and cobalt restored the activity of TMCP2 in a dose-dependent manner (fig. S5E). This revealed a marked difference between the two enzymes, as TMCP1 was reactivated by cobalt and manganese but not zinc (fig. S2E).

### β-Tubulin tail processing activity of TMCP2

Notably, the overexpression of active TMCP2 isoforms also generated a signal recognized by α∆2 antibodies in the β-tubulin region (Fig. 4, B and C). This result implies that TMCP2, apart from processing α-tubulin, also modifies β-tubulin. The epitope recognized by α∆2 antibodies corresponds to -EEEGE sequence, which is highly similar to motifs present within the tail of βI-, βII-, and βIV-tubulin (Fig. 4F). Thus, we generated a series of C-terminally truncated βI- and βIV-tubulins and found that α∆2 antibodies recognized exclusively the βI-tubulin missing the last three (-EEA) and βIV-tubulin missing the last four (-EEVA) amino acids (fig. S6A) that closely resemble the consensus sequence. Because HEK293 cells express predominantly βI- and βIV-tubulin isotypes (*40*), it suggests that TMCP2 apart from generating α∆2 also catalyzes βIΔ3 and/or βIV∆4 modification(s). Mass spectrometry analysis of tubulin purified from cells overexpressing active TMCP2 (fig. S7A) detected a slight increase in the levels of α∆1-tubulin and an accumulation of α∆2 modification (fig. S7B), which was consistent with enzymatic specificity of TMCP2 on α-tubulin (Fig. 4, B to E). Furthermore, we observed an increase in the levels of βIΔ3-tubulin while βIV-tubulin remained unmodified (fig. S7C). This suggests that TMCP2 apart from α-tubulin specifically modifies βI- but not βIV-tubulin.

**Fig. 5.**
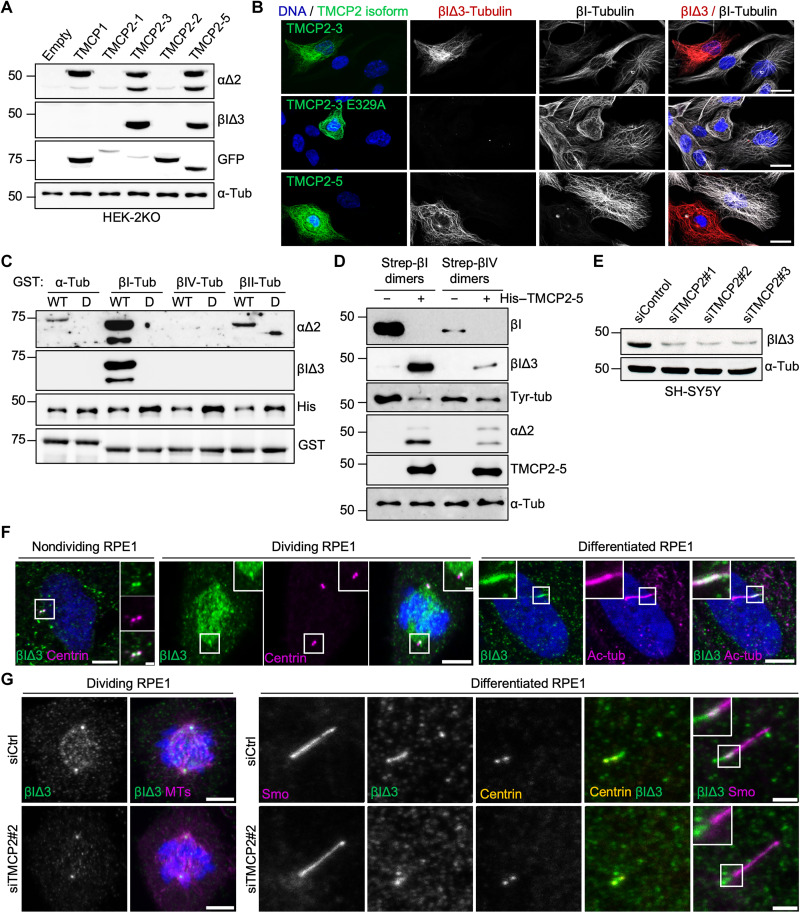
TMCP2 catalyzes previously unknown βIΔ3 modification. (**A**) Immunoblots of protein extracts from HEK-2KO cells expressing TMCP1 and several isoforms of TMCP2 probed with the indicated antibodies, including the newly generated anti–βIΔ3-tubulin antibody. (**B**) Immunofluorescence analysis of RPE1 cells expressing TMCP2-3 or its enzymatically inactive version (E329A) and TMCP2-5 using anti–βI-tubulin or anti–βIΔ3 antibody. Scale bars, 20 μm. (**C**) Immunoblots of an in vitro assay involving recombinant TMCP2-5 or its enzymatically inactive version and bacterially produced GST-α-, βI-, βII-, and βIV-tubulin. (**D**) Immunoblot analysis of an in vitro assay involving recombinant TMCP2-5 and tubulin dimers enriched for βI- or βIV-tubulin after Strep-tag–based purification from HEK293 cells. (**E**) Immunoblots of protein extracts from SH-SY5Y cells following knockdown of *TMCP2* using three different siRNAs. (**F**) Immunofluorescence analysis of endogenous βIΔ3-tubulin in nondividing, mitotic, or differentiated RPE1 cells. βIΔ3-tubulin is enriched at centrioles and mitotic spindles as well as primary cilia. Scale bars, 5 μm; inset, 1 μm. (**G**) Immunofluorescence analysis of either dividing or differentiated RPE1 cells knockdown for *TMCP2*. Scale bars, 5 μm for dividing RPE1 and 2 μm for differentiated RPE1.

To confirm that TMCP2 indeed catalyzes βI∆3 modification, we developed a new antibody against modified βI-tubulin (for details, see Materials and Methods). The specificity of the antibody has been initially tested using series of C-terminally truncated βI- and βIV-tubulin. As expected, the antibody recognizes exclusively βI∆3- and βIV∆4-tubulin epitopes (fig. S6B). To assess the β-tubulin processing activity of TMCPs, we performed immunoblot analysis using extracts from HEK293 cells expressing either TMCP1 or various TMCP2 isoforms. We detected a single band in the β-tubulin region only in the extracts from cells expressing active TMCP2 isoforms (Fig. 5A). These results were further validated by immunofluorescence analysis, which showed MT-associated labeling by βI∆3 antibodies exclusively in cells expressing active but not inactive TMCP2 isoforms (Fig. 5B). Together, these data confirm that TMCP2 generates βI∆3 modification.

Next, we set out to test the enzymatic preference of TMCP2 either toward α-, βI-, βII-, or βIV-tubulin using bacterially produced substrates. As demonstrated by immunoblot with α∆2-tubulin antibody, the preferred substrate of TMCP2 is βI-tubulin, which was modified much more efficiently than α- or βII-tubulin, while βIV-tubulin remained unmodified (Fig. 5C). The enzymatic preference of TMCP2 was further confirmed by labeling with βI∆3 antibodies, which recognized exclusively βI- but not βIV-tubulin treated with an active enzyme (Fig. 5, C and D).

To further validate our results obtained with bacterially produced substrates, we engineered βI- and βIV-tubulin with a Strep-tag inserted in the internal loop close to the N terminus (fig. S6E). Upon polymerization, this part of the tubulin is predicted to localize to the MT lumen, and thus, the presence of the tag should not interfere with MT assembly. Immunofluorescence analysis of the cells expressing Strep-tagged tubulins showed their incorporation into the MTs (fig. S6F), demonstrating that they are indeed polymerization competent and thus likely to be properly folded. To further test the enzymatic preference of TMCP2, we used Strep-tagged βI-tubulin– and βIV-tubulin–based dimers purified from HEK293 cells as a substrate. The efficiency of modification was monitored by an antibody, which recognizes an epitope in the very C terminus of βI-tubulin (*41*) and as such is sensitive to the removal of even a single amino acid (fig. S6C). We found that βI-tubulin antibody detected a signal exclusively in the untreated sample, while βI∆3 antibody recognized a band in the sample incubated with TMCP2 (Fig. 5D and fig. S6D), indicating a complete processing of the βI-tubulin tail. A trace amount of the βI∆3 signal was also found in the βIV-tubulin treated with TMCP2 (Fig. 5D). It most likely represented a modified βI-tubulin due to the slight contamination present in the βIV-tubulin preparation, as shown with βI-tubulin signal (Fig. 5D). Last, the analysis with α∆2 antibodies provided further evidence that even in the presence of α-tubulin, βI-tubulin is the preferred substrate of TMCP2 (Fig. 5D).

Subsequently, we tested whether the removal of the C-terminal amino acids from βI-tubulin is also sequential, as was the case for α-tubulin. We evaluated the activity of TMCP2 using either the WT βI-tubulin or its shorter derivatives lacking one or two amino acids. We found that all three substrates were modified (fig. S6G), suggesting that the processing of β-tubulin by TMCP2 is indeed sequential.

Next, we examined whether the aspartate present within the C terminus of βII-tubulin might be inhibitory. We compared the activity of TMCP2 toward the WT βII-tubulin or its mutated version, in which the aspartate has been replaced by a glutamate (-GEDEA versus -GEEEA). We found that the processing of mutant βII-tubulin was strongly increased (fig. S6H), demonstrating that the presence of aspartate in the sequence of the βII-tubulin C terminus inhibits the activity of TMCP2.

### Subcellular localization of the βI∆3 modification

Having shown that TMCP2 modifies βI-tubulin, we tested four human cell lines of different origins including RPE1, HEK293, U2OS, and SH-SY5Y for the presence of the endogenous βI∆3 modification. Immunoblot analysis detected a specific signal in the β-tubulin region in all cell lines tested (fig. S8A). Moreover, depletion of TMCP2 in SH-SY5Y cells with three different siRNAs led to a strong reduction in the βI∆3 signal on immunoblot (Fig. 5E and fig. S8B), showing that this enzyme is responsible for the generation of endogenous βI∆3 modification.

Next, we evaluated cellular distribution of the endogenous βI∆3-tubulin. Using immunofluorescence analysis, we detected the presence of βI∆3 on centrioles and mitotic spindles in various cell lines including RPE1, U2OS, and SH-SY5Y (Fig. 5F and fig. S8, C and E). In addition, we found that βI∆3-specific antibody also labels the base of the cilia in differentiated RPE1 and Madin-Darby canine kidney (MDCK) cells (Fig. 5F and fig. S8D). The specificity of the signal was confirmed by the *TMCP2* knockdown experiments, which led to a strong reduction in the level of ciliary and spindle βI∆3-tubulin (Fig. 5G and fig. S8E).

### Tubulin deglutamylase activity of TMCP1 and TMCP2

**Fig. 6.**
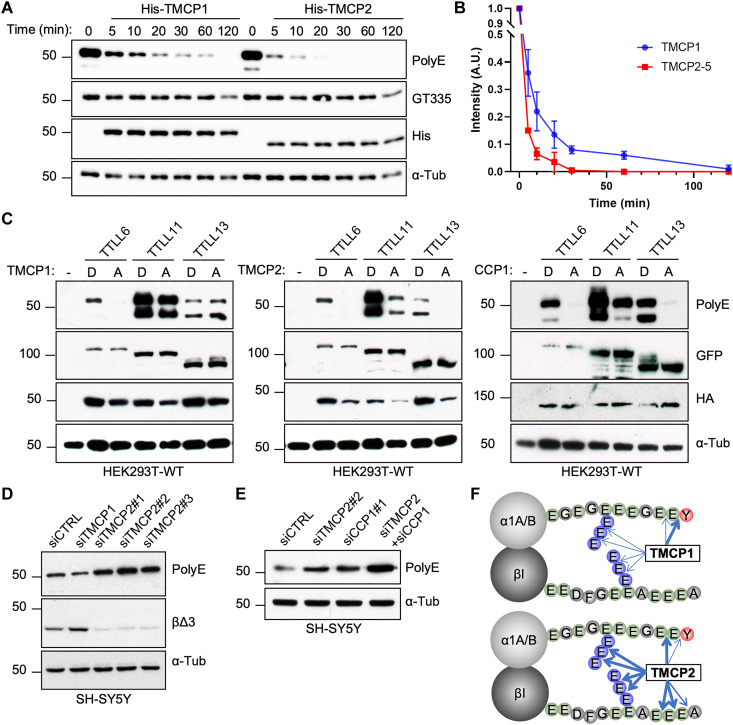
TMCPs catalyze the removal of posttranslational polyglutamylation. (**A**) Immunoblots of an in vitro deglutamylation assay measuring time-dependent activity of recombinant His-TMCP1 and His-TMCP2 toward MTs derived from mouse brain. (**B**) Graphical representation of His-TMCP1 and His-TMCP2 deglutamylase activities. Immunoblot signals (polyE antibody) were quantified for each time point (mean ± SD; *n* = 3 independent experiments). (**C**) Immunoblots of protein extracts from HEK293 cells coexpressing GFP-TTLL6, GFP-TTLL11, or GFP-TTLL13 in the presence of either active or enzymatically dead HA-TMCP1, HA-TMCP2, and HA-CCP1. (**D**) Immunoblots of protein extracts from SH-SY5Y cell knockdown for *TMCP1* or *TMCP2*. Three different siRNAs were used for *TMCP2*, one siRNA for *TMCP1*, and a scramble siRNA as control. (**E**) Immunoblots of protein extracts from SH-SY5Y cells following knockdown of *TMCP2* or *CCP1*, either alone or in combination. (**F**) Schematic overview of α- and β-tubulin modifications catalyzed by TMCP1 and TMCP2. The width of the arrows represents the cleavage efficiency.

Because both TMCPs removed the C-terminally exposed glutamates, as demonstrated by their ability to generate α∆2 modification, we tested whether they can also remove posttranslational polyglutamylation. We set up an in vitro deglutamylation assay using highly polyglutamylated brain tubulin as a substrate combined with recombinant TMCPs. The samples were analyzed with two glutamylation-specific antibodies, polyE which recognizes long glutamate chains composed of at least three glutamates and GT335 that specifically detects the branching point glutamate regardless of the chain length (*19*). As demonstrated by immunoblot analysis, both TMCPs reduced the level of polyE but not GT335 in a time-dependent manner, with TMCP2 being slightly more active as compared to TMCP1 (Fig. 6, A and B). This suggests that TMCPs have the ability to shorten posttranslationally added glutamate chains but not to remove the branching point glutamates, which is highly reminiscent of the enzymatic specificity of CCP1 (*17*). To further assess deglutamylase activity of TMCPs, we set up a competition experiment in which either active or inactive TMCPs were coexpressed with TTLL6, TTLL11, and TTLL13, the three autonomously active polyglutamylases (*19*). CCP1 deglutamylase was used as a positive control. We observed that similarly to CCP1, active TMCP2 efficiently inhibited the accumulation of polyglutamylation generated by all three enzymes (Fig. 6C). Conversely, TMCP1 blocked the activity of TTLL6, was less efficient with TTLL11, and had no effect on TTLL13 (Fig. 6C). This suggests that TMCP2 is a highly active deglutamylase comparable to CCP1, while TMCP1 is less efficient although it can remove certain type of posttranslational polyglutamylation.

Next, we tested whether endogenous TMCP1 and TMCP2 are associated with deglutamylation. We found that the knockdown of *TMCP1* in SH-SY5Y cells did not increase the level of polyglutamylation (Fig. 6D). In contrast, depletion of *TMCP2* with three different siRNAs led to a strong accumulation of polyglutamylation, which was comparable to *CCP1* depletion (Fig. 6, D and E, and fig. S8F). Furthermore, codepletion of *TMCP2* and *CCP1* had an additive effect resulting in further increase in the amount of polyglutamylation (Fig. 6E), suggesting that both enzymes are involved in controlling the level of polyglutamylation, at least in SH-SY5Y cells.

In summary, TMCP1 is a highly efficient detyrosinase, which can also generate α∆2 modification and remove certain types of posttranslational polyglutamylation although less efficiently than TMCP2. In contrast, TMCP2 appears to preferentially modify β-tubulin and to be a highly efficient deglutamylase with activity comparable to CCP1 (Fig. 6F).

### Evolutionary conservation of the TMCP enzymes

**Fig. 7.**
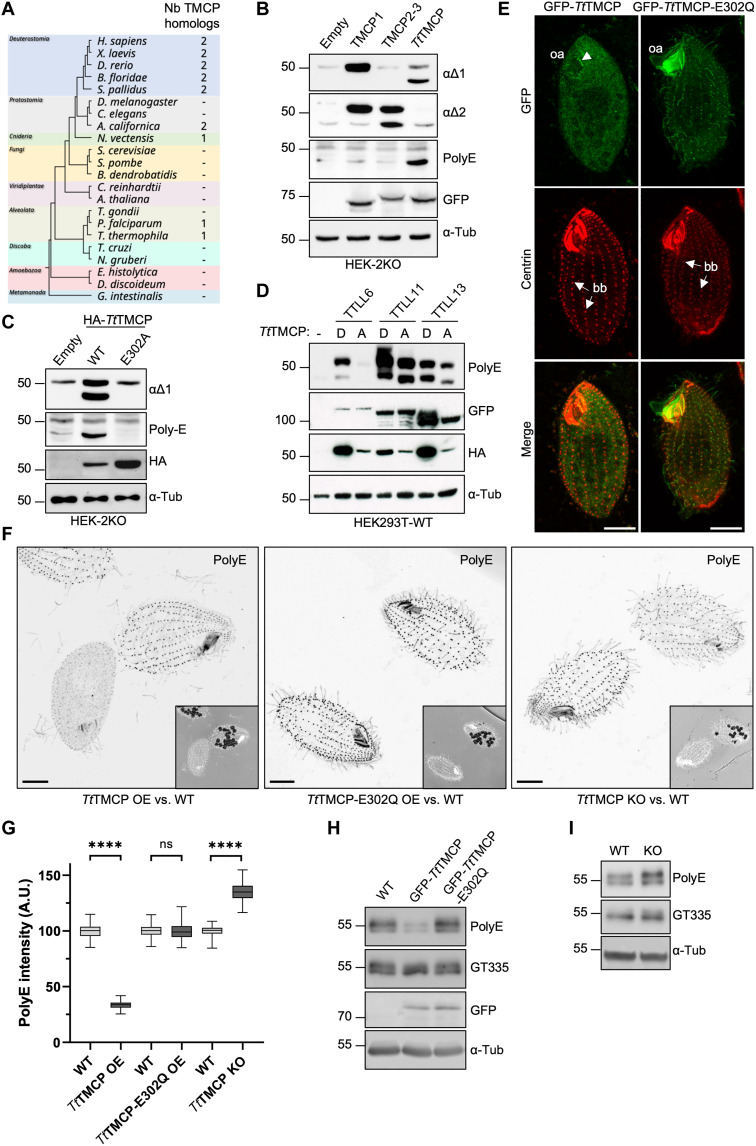
Molecular and functional analysis of a TMCP homolog from *Tetrahymena thermophila*. (**A**) Taxonomic tree of selected eukaryotes depicting the conservation of TMCPs. (**B**) Immunoblots of protein extracts from HEK-2KO cells expressing either GFP-TMCP1, GFP-TMCP2-3, or GFP-*Tt*TMCP (*Tetrahymena thermophila* TMCP). (**C**) Immunoblots of protein extracts from HEK-2KO cells expressing *Tt*TMCP or its enzymatically inactive version (E302A). (**D**) Immunoblots of protein extracts prepared from HEK293 cells coexpressing GFP-TTLL6, GFP-TTLL11, or GFP-TTLL13 together with either active or enzymatically dead HA-*Tt*TMCP. (**E**) Immunofluorescence analysis of *Tetrahymena* cells showing the localization of GFP-*Tt*TMCP or its enzymatically inactive version (E302Q) upon overexpression. Arrows are pointed at somatic basal bodies (bb), while the arrowhead indicates the basal bodies in the oral apparatus (oa). (**F**) Immunofluorescence analysis of *Tetrahymena* cells labeled with polyE antibody upon expression of either GFP-*Tt*TMCP or GFP-*Tt*TMCP-E302Q and the *TtTMCP* knockout cells. Comparison is made with WT cells on the same coverslip, which are identified by the presence of ink vacuoles. Scale bars, 10 μm. (**G**) Graphical representation of polyE signal intensity on the basal bodies from the experiments represented in (F) (****P < 0.0001 calculated with nested *t* test). ns, not significant. (**H**) Immunoblots of protein extracts from *Tetrahymena* cells expressing GFP-*Tt*TMCP or its enzymatically inactive version E302Q as compared to WT. (**I**) Immunoblots of protein extracts from *Tetrahymena* cells knockout for *TtTMCP* as compared to WT.

Given the existence of two TMCP homologs with different specificities in humans, we set out to determine which type of enzyme appeared first in the evolution. Thus, we examined the evolutionary distribution of TMCPs. The survey of 22 evolutionary divergent organisms showed that among single-cell eukaryotes, TMCP homologs are found exclusively in certain members of alveolate, including *Tetrahymena* and *Plasmodium* but unexpectedly are absent from *Toxoplasma*. Furthermore, fungi and plants also lack TMCP homologs (Fig. 7A), while a single TMCP homolog is present in *Nematostella*, a representative of cnidaria. Notably, two TMCP homologs were found in deuterostomia and primitive protostomia suggesting a gene duplication event in the ancestor of bilateralia. No homologs of TMCP were found in *Drosophila* and *Caenorhabditis elegans* despite the presence of detyrosination (*42*, *43*). Considering that insects and nematodes also lack VASH homologs (*39*), this was highly unexpected and suggests the existence of yet another enzyme involved in tubulin detyrosination.

Because among single-cell organisms TMCP homologs have been found exclusively in alveolata, we decided to characterize the enzymatic properties and the biological role of the TMCP homolog from *Tetrahymena thermophila* (*Tt*TMCP). Immunoblot analysis of HEK-2KO cells overexpressing active but not catalytically dead *Tt*TMCP showed the appearance of prominent polyE and α∆1 signal in the β-tubulin region (Fig. 7, B and C). Considering the specificity of the antibodies, this suggests that *Tt*TMCP modifies preferentially β-tubulin by the removal of either alanine alone (polyE signal) or alanine followed by a glutamate (α∆1 signal). Furthermore, these results provide additional evidence for sequential processing of the β-tubulin by TMCPs.

Next, we evaluated deglutamylase activity of *Tt*TMCP by coexpressing it with autonomously active human polyglutamylases, TTLL6, TTLL11, and TTLL13 in HEK293 cells. We found that the expression of active *Tt*TMCP reduced the accumulation of polyglutamylation generated by all three enzymes (Fig. 7D). Together, these results suggest that *Tt*TMCP appears to share more characteristics with human TMCP2 than TMCP1.

To further validate this conclusion, we overexpressed either active or inactive *Tt*TMCP in *Tetrahymena*. The active enzyme showed mostly cytosolic localization; however, it was also found to be weakly associated with the basal bodies of the oral apparatus (Fig. 7E). In contrast, the enzymatically dead *Tt*TMCP colocalized with most basal bodies and was also found in cilia (Fig. 7E and fig. S9A). Stronger association of the inactive *Tt*TMCP with the MT-based structures is consistent with data obtained for enzymatically dead human TMCPs, which also showed stronger binding to MTs as compared to their active counterparts (Figs. 2C and 4D). Immunofluorescence analysis showed that overexpression of the active but not inactive *Tt*TMCP led to a strong reduction of the polyE signal specifically on the basal bodies (Fig. 7, F and G), while the GT335 signal remain unchanged (fig. S9, B and C). These findings were further validated by immunoblot analysis (Fig. 7H), suggesting that *Tt*TMCP similarly to human TMCPs can shorten the glutamate chain but does not remove the branching point glutamate.

Last, to determine the functional importance of *Tt*TMCP, we developed knockout *Tetrahymena* cells using a codeletion method (fig. S10A). We obtained several mutants with different size of deletion of the *TtTMCP* locus (fig. S10B) and confirm the lack of *TtTMCP* expression in two independent mutants carrying the largest deletion using reverse transcription polymerase chain reaction (RT-PCR) (fig. S10C). Because proliferation assays showed that both knockout strains grew similarly to WT cells (fig. S10D), for further examination, we used only one of them (KO-1). Immunofluorescence analysis of the knockout cells showed an increase in the level of polyE but not GT335 labeling on the basal bodies (Fig. 7, F and G, and fig. S9, B and C). The overall increase of the polyE signal in the *TtTMCP* knockout cells has been further confirmed by immunoblot analysis (Fig. 7I). In summary, *Tt*TMCP is a tubulin deglutamylase that regulates the level of tubulin polyglutamylation specifically on the basal bodies. Furthermore, considering that *Tt*TMCP specifically modifies β-tubulin when expressed in mammalian cells, it is highly unlikely to be involved in catalyzing α-tubulin detyrosination in *Tetrahymena* cells.

## DISCUSSION

### Identification of a previously unknown tubulin detyrosinase

The discovery of VASHs as the first class of enzymes involved in detyrosination revealed the existence of additional enzyme(s) catalyzing this modification (*14*). Therefore, the original objective of our study was the identification of the missing detyrosinase(s) in mammalian cells. Here, we describe the discovery of TMCP1 as a second class of enzymes involved in the generation of αΔ1-tubulin. We show that depletion of TMCP1 in knockout cells lacking VASHs or in WT cells treated with VASH inhibitor almost completely abolishes detyrosination. Hence, our results show that these two types of enzymes are the major mammalian detyrosinases. In agreement, recent study which independently identified TMCP1 as tubulin detyrosinase, showed that the brains of double-knockout mice lacking *SVBP* and *TMCP1* are almost completely devoid of detyrosination (*32*). Considering that neurons express high levels of α4-tubulin, a tubulin isotype that lacks the very C-terminal tyrosine residue, these results are rather unexpected. One possibility is that in the absence of detyrosinating enzymes, the unopposed TTL activity efficiently retyrosinates α4-tubulin. In support of this hypothesis, a study involving mass spectrometry analysis of brain tubulin has identified peptides corresponding to tyrosinated α4-tubulin (*44*). In addition, a similar analysis of liver tubulin showed that α4-tubulin in this tissue is fully tyrosinated (*45*). Together, this suggests that TTL is highly efficient at modifying α4-tubulin, which in the absence of detyrosination might undergo complete retyrosination.

Furthermore, although TMCP1 has a strong detyrosinase activity, we find that it also catalyzes αΔ2 modification. Until now, the formation of the αΔ2-tubulin was believed to be a two-step process involving different types of enzymes: VASHs, which remove the tyrosine, and CCPs, which cut off the penultimate glutamate. Our demonstration that TMCP1 alone can catalyze αΔ2-tubulin further underlines the potential importance of this rather understudied modification. This becomes particularly relevant in the light of the recent study showing that abnormal accumulation of αΔ2-tubulin is associated with neurodegeneration (*46*). Last, considering that the homologs of VASHs and TMCPs are absent from *Drosophila* and *C. elegans*, two model organisms with documented presence of detyrosination (*42*, *43*), this suggests the existence of yet another tubulin detyrosinase that remains to be identified.

### Discovery of a β-tubulin–specific posttranslational modification

The C-terminal tail of α-tubulin is subjected to a sequential removal of up to three amino acids, which results in the generation of either αΔ1-, αΔ2-, or αΔ3-tubulin, respectively (*1*, *18*, *47*). Here, we show that similar processing occurs on β-tubulin. In contrast to the C-terminal tails of α-tubulin, which share a high degree of homology between different isotypes, β-tubulin tails are rather divergent (*48*). This implies that an enzyme involved in the processing of β-tubulin is likely to act only on a limited number of isotypes. In agreement with this hypothesis, we show that the main substrate of TMCP2 is βI-tubulin, which undergoes sequential removal of three amino acids leading to the formation of βIΔ3 modification. We did not observe intermediate isoforms such as βIΔ1 or βIΔ2. This is most likely due to the enzymatic characteristics of TCMP2, which is highly efficient at the removal of alanine and glutamate. In support of this hypothesis, partially processed βI-tubulin species were detected upon overexpression of the *Tetrahymena* TMCP, which appears to be less active when expressed in mammalian cells.

Using modification-specific antibodies, we detected endogenous βIΔ3-tubulin in various cell lines derived from different origins, demonstrating that it is a widely spread modification. At the cellular level, βIΔ3 antibodies label mitotic spindles and centrioles, suggesting a potential role of this modification in cell division. In addition, we show the presence of βIΔ3 modification in the primary cilia. Similar modification has been previously described in sperm flagella from Sea urchin (*Strongylocentrotus* *pallidus*), an organism containing two TMCP homologs, where around 20% of the main β-tubulin isotype lacked the last three amino acids (-GDEEAA versus -GDE) (*49*). Notably, the truncated tubulin was found to be also highly polyglycylated, raising the possibility that β-tubulin processing might regulate the activity of enzymes involved in polymodifications.

Among other β-tubulins, the two isotypes with the most similar C-terminal tails to βI-tubulin are βII and βIV. We have found that βIV-tubulin is not modified by TMCP2, while weak activity has been detected on βII-tubulin. The efficiency of the βII-tubulin modification was strongly increased by replacing the aspartate residue with glutamate (-GEDEA versus -GEEEA), which suggests that the presence of aspartate strongly reduces the activity of TMCP2. Considering that CCP3 has been previously shown to catalyze the removal of aspartates with similar efficiency as glutamates (*21*), it raises the possibility that the two types of enzymes might cooperate in modifying βII-tubulin. A mass spectrometry–based study involving brain tubulin has identified a truncated version of βII-tubulin missing the last four amino acids, which has been termed βIIΔ4-tubulin (*18*). Because the activity of CCPs is restricted to acidic residues and does not catalyze the removal of alanine (*18*), it implies that the modification of βII-tubulin is under the control of TMCP2, which is absolutely required to initiate the processing. Once the alanine is removed, CCPs are likely to be involved in the removal of acidic residues, in particular the aspartate that can be efficiently cut off only by CCP3 (*21*). Moreover, because to generate αΔ3-tubulin CCPs remove a glutamate from a glycine residue, they might also be able to convert βIIΔ3-tubulin into βIIΔ4-tubulin, accounting for the presence of this isoform in the brain (*18*). Currently, it remains unknown whether CCPs can also remove glutamates from alanine and thus convert βIΔ3-tubulin into βIΔ4-tubulin. Together, our work shows that the C termini of some β-tubulins are subjected to a processing previously thought to be restricted to α-tubulins. Furthermore, it implies the existence of specific readers being able to recognize modified β-tubulin. The identification of such protein(s) is likely to be the focus of future research in this field.

### Removal of posttranslational polyglutamylation by TMCPs

The existence of regulatory mechanisms involved in controlling the levels of tubulin polyglutamylation have been already proposed soon after its discovery (*50*). In recent years, the members of the TTLL family have been identified as the forward enzymes adding the glutamate chains, while the members of the CCP family were shown to carry out the reverse reaction. This led to a model, in which the level of polyglutamylation in a particular cell is established as a competition between these two types of enzymes. Previously, we have shown that deglutamylation is a two-step process with most CCPs catalyzing the shortening of the glutamate chain, while CCP5 is the only enzyme capable of removing the branching point glutamate (*17*). Our experiments show that the deglutamylase activity of TMCPs closely resembles that of CCP1 and that both enzymes collectively control the level of polyglutamylation, at least in the SH-SY5Y cells. Considering that abnormal accumulation of tubulin polyglutamylation has highly deleterious effects resulting in various ciliopathies and neurodegeneration (*17*, *31*), the identification of another class of enzymes associated with this modification adds yet another layer of control.

### Regulatory mechanism of the TMCP activity

Although at the start of our study TMCPs were not annotated as enzymes, we show that they are metalloproteases. Their structure, which was initially predicted by AlphaFold (*35*) and later solved experimentally (*32*), indicates that they belong to a clan of gluzincin metallopeptidases (*36*). The members of this clan are generally characterized by the presence of HExxH motif, which is involved in metal coordination. The two His residues of this motif contribute to the coordination of divalent metal ions, in most cases Zn^2+^ but rarely also other metals such as Mn^2+^ and Co^2+^. In case of TMCPs, the HExxH motif is extended to HExxxH, which, although unusual, has already been found in other metallopeptidases (*51*, *52*). The most studied enzyme harboring such motif is dipeptidyl peptidase III (DPP III), which is a Zn^2+^-dependent aminopeptidase. It hydrolyses sequential removal of dipeptides from the N terminus and is involved in protein turnover (*53*). In contrast to DPP III, TMCPs are carboxypeptidases that sequentially remove single amino acids from the C terminus. In addition, similarly to DPP III, TMCP2 is also activated by Zn^2+^. In contrast, this metal is rather inhibitory for TMCP1. Instead, TMCP1 is strongly activated by Co^+2^ and Mn^+2^, which also activate TMCP2 similarly to Zn^2+^. Although the structural study showed the presence of Zn^2+^ in the active site of TMCP1, it might not represent the actual metal bound by this enzyme in physiological conditions as crystallization buffer was specifically supplemented with Zn^2+^ (*32*). Therefore, more detailed analysis is required to unequivocally establish which metal activates TMCP1.

Furthermore, we show that although TMCP1 preferentially modifies MTs, it can also process tubulin dimers. Similar specificity has been observed for VASH1, while VASH2 displayed unequivocal preference for MTs (*39*). Overall, both types of enzymes preferentially modify the polymer, which is consistent with accumulation of detyrosination on highly stable MTs.

In addition, our work reveals an interesting regulatory mechanism specific to TMCP2, which involves alternative splicing of exon 5. TMCP2 isoforms, in which the exon 5 has been retained, are active, while the isoforms lacking this exon are inactive. We show that the part of the protein encoded by exon 5 is required for MT binding by TMCP2. Our findings are strongly supported by cryo–electron microscopy study, which showed that corresponding sequence in TMCP1 encodes a domain involved in binding to helix 12 of α-tubulin located on the MT surface (*32*). Hence, this interaction is likely to play an essential role in positioning TMCP1 in the vicinity of the C-terminal tail of α-tubulin. Because TMCP2 isoforms lacking exon 5 retain the active site, it raises the question of whether they might still be active but only toward potential nontubulin substrates. This is particularly interesting in the case of the canonical TMCP2 isoform, which contains a nuclear localization signal (NLS) and localizes to the nucleus. It suggests that perhaps TMCP2 modifies the primary chain of nontubulin substrates located in the nucleus or, alternatively, it might act as a reverse enzyme for numerous nuclear substrates of polyglutamylation, which have already been identified (*54*). Future studies will determine whether TMCP2 has indeed additional substrates.

In conclusion, we found a family of tubulin-modifying enzymes composed of two members, TMCP1 and TMCP2, which catalyze the processing of α- and β-tubulin C-terminal tails. TMCP1 is specific to α-tubulin and acts as an efficient detyrosinase with activity similar to VASHs. However, unlike VASHs, TMCP1 also generates αΔ2 modification through sequential removal of tyrosine and glutamate. As for TMCP2, although it can modify α-tubulin upon overexpression, its physiologically relevant substrate is βI-tubulin from which it sequentially removes three residues generating previously unknown βIΔ3 modification. By using a newly developed modification-specific antibody, we show that βIΔ3-tubulin is present on centrioles, mitotic spindles and cilia in various cell lines of different origin, suggesting that it may play a role in cell division and ciliogenesis.

Moreover, we demonstrate that TMCPs also remove posttranslational polyglutamylation acting as tubulin deglutamylase with activity similar to CCP1. In agreement, we further show that TMCP homolog from *Tetrahymena*, an evolutionary distant organism, is also a tubulin deglutamylase specifically involved in the regulation of polyglutamylation at the basal bodies.

In the future, deciphering the precise function(s) of the β-tubulin processing will be of crucial importance. This is also the case for the abundant neuronal α∆2-tubulin, for which specific functions remain to be identified.

## MATERIALS AND METHODS

### Cell culture and transfection

HEK293T cells, WT or knockout for *VASH1* and *VASH2* (HEK-2KO) (*17*), as well as RPE1 and MDCK cells, were cultured in Dulbecco’s modified Eagle’s medium/F-12 GlutaMAX-I (Gibco) supplemented with 10% heat-inactivated fetal bovine serum (Gibco) and antibiotics (penicillin/streptomycin) (Gibco). U2OS and SH-SY5Y cells were cultured in Dulbecco’s modified Eagle’s medium + GlutaMAX-I (Gibco) supplemented with 10% heat-inactivated fetal bovine serum (Gibco) and 1% penicillin/streptomycin (Gibco). For all cell lines, transfection of expression plasmids was achieved with JetPEI transfection reagent (Polyplus) according to manufacturer’s guidelines. Knockdowns of *TMCP1*, *TMCP2*, and *CCP1* were performed with predesigned Dicer-Substrate Short Interfering RNAs from Integrated DNA Technologies (TriFECTa Kit from IDT), which were transfected into corresponding cells with lipofectamine RNAiMAX (Thermo Fisher Scientific) according to the manufacturer’s guidelines. Cells were collected 24 to 48 hours after transfection (or 72 hours for knockdown experiments) for biochemical and immunofluorescence analyses. References of the DsiRNA are hs.Ri.KIAA0895L.13.1, hs.Ri.KIAA0895L.13.2 and hs.Ri.KIAA0895L.13.3 for *TMCP1*; CD.Ri.386658.13.1, hs.Ri.KIAA0895.13.3 and hs.Ri.KIAA0895.13.1 for *TMCP2*; and hs.Ri.AGTPBP1.13.1, hs.Ri.AGTPBP1.13.2, and hs.Ri.AGTPBP1.13.3 for *CCP1*.

### In vitro detyrosination activity assay from cell lysates

HEK-2KO cells were harvested and lysed in 80 mM Pipes (pH 6.8), 5 mM MgCl_2_, and 0.2% NP-40. The suspension was rotated for 30 min at 4°C before centrifugation at 15,000*g* for 10 min. The cleared supernatant was incubated at 37°C for 2 hours (detyrosination activity stimulation). For some experiments, cell extracts were supplemented with either protease inhibitors or metal divalent cation salts (ZnCl_2_, MnCl_2_, and CoCl_2_ at 2 mM). The different inhibitors and working concentrations used throughout the study were the following: pepstatin (Thermo Fisher Scientific, 2 μM), phenylmethylsulfonyl fluoride (Sigma-Aldrich, 1 mM), leupeptin (Sigma-Aldrich, 50 μM), iodoacetamide (Sigma-Aldrich, 10 μM), *N*-ethylmaleimide (Sigma-Aldrich, 20 mM), E-64 (Thermo Fisher Scientific, 50 μM), Epoxide-Y (EpoY) (Sigma-Aldrich, 50 μM), EDTA (Sigma-Aldrich, 1 mM), EGTA (Sigma-Aldrich, 1 mM), 1,10-phenanthroline (Sigma-Aldrich, 1 mM), dl-benzylsuccinic acid (BSA) (Sigma-Aldrich, 1 mM), 2-(phosphonomethyl)-pentanedioic acid (Sigma-Aldrich, 1 mM) and 2-guanidinoethylmercaptosuccinic acid (Abcam, 1 mM).

### Biochemical enrichment of detyrosination activity

To identify the remaining tubulin-detyrosinating enzyme(s), we designed a two-step purification procedure from HEK-2KO cells. All steps were performed at 4°C. Around 1 billion cells (the equivalent of 20-mm by 150-mm confluent dishes) were resuspended in one volume of 50 mM Pipes buffer at pH 6.8, 0.2% NP-40, 10% glycerol, and protease inhibitor cocktail (Roche). Cell lysis was completed by Dounce homogenization, and the suspension was rotated for 20 min before centrifugation at 15,000*g*. The clarified supernatant was further centrifuged at 100,000*g* for 30 min on a Beckman Optima TLX-120 Ultracentrifuge. The resulting supernatant was collected and used as a protein extract for the purification procedure. As a first purification step, we used size exclusion chromatography. The protein extract was applied to a Superose 6 gel filtration column (HR 10/30 GE Healthcare) using a 500-μl sample loading loop on an Äkta purifier system (GE Healthcare) and resolved in 50 mM Pipes buffer at pH 6.8 and 10% glycerol at a flow rate of 0.3 ml min^−1^. Fractions (0.5 ml) were collected, and the procedure was repeated three times in total. Calibration of the Superose 6 column was performed with protein standards of known molecular weights using the Gel Filtration Calibration Kit (GE Healthcare). The matching fractions between the three column runs were pooled, and an aliquot (50 μl) was analyzed for detyrosination activity after addition of 2 mM MnCl_2_ and incubation at 37°C for 1 hour. An input corresponding to 1% of protein extract loaded onto the column was included as a positive control on the same immunoblot. The detyrosination activity peaked between fractions 28 and 32, which also corresponded to the elution peak of free tubulin. Fractions 28 to 32 were thus pooled (⁓7.5 ml in total), concentrated, and buffer exchanged in 50 mM Pipes buffer at pH 6.8 (without glycerol) using 10-kDa Amicon centrifugal filter units (Amicon Ultra-4 Merck Millipore). The concentrated protein sample was used for the second purification step. The sample was loaded onto a Mono S 5/50 cation-exchange chromatography column (GE Healthcare) using a 500-μl sample loading loop on an Äkta purifier system (GE Healthcare). The column had been equilibrated with 50 mM Pipes buffer at pH 6.8 as the binding buffer (buffer A). After sample injection and binding, the column was washed with 10 ml of buffer A. Bound proteins were then eluted over a linear gradient of buffer B (buffer A with 500 mM KCl) with increasing ionic strength from 0 to 100% buffer B. Twenty fractions of 1 ml were collected at a flow rate of 0.5 ml min^−1^ for 40 min. The fractions were then concentrated and buffer exchanged in buffer A to remove salts using 10-kDa Amicon tubes (Amicon Ultra-0.5 Merck Millipore) (final volume, ⁓50 μl per fraction). To test for the detyrosination activity after elution, an aliquot of each fraction (15 μl) was incubated in the presence of 1-μg recombinant GST–α-tubulin as a reaction substrate and supplemented with 1 mM MnCl_2_ in a reaction buffer (50 mM Pipes buffer at pH 6.8 and 10% glycerol) (final volume, 50 μl). The reaction was done overnight, and fractions 7 to 20 were immunoblotted for detyrosinated GST–α-tubulin. The detyrosinase activity peaked between fractions 15 and 17, which correspond to a salting out of ⁓400 mM KCl.

### Mass spectrometry sample preparation and analysis

On the basis of the elution profile of the detyrosinase activity after Mono S chromatography, two fractions were chosen for mass spectrometry analysis. These included fractions 11, which had no detectable activity (negative control), and fraction 15 corresponding to detyrosinase activity peak. Seventy-nine microliters of 100 mM triethylammonium bicarbonate buffer (TEAB) was added to each sample (10 μl). Samples were incubated for 30 min at 60°C after addition of 1 μl of 1 M dithiothreitol. A volume of 10 μl of 0.5 M iodoacetamide was added (incubation for 30 min in the dark). Enzymatic digestion was performed by addition of 1 μg of trypsin in TEAB 100 mM and incubation overnight at 30°C. Peptides were desalted using OMIX Tips (Agilent Technologies Inc.) according to the manufacturer’s specifications and then were dehydrated in a vacuum centrifuge. Peptides were analyzed online by nanoflow high-performance liquid chromatography (HPLC)–nanoelectrospray ionization using a Q Exactive HFX mass spectrometer (Thermo Fisher Scientific) coupled to a nano–liquid chromatography (nano-LC) system (U3000-RSLC, Thermo Fisher Scientific). Desalting and preconcentration of samples were performed on-line on a Pepmap precolumn (0.3 mm by 10 mm, Dionex). A gradient consisting of 0 to 40% B in A for 120 min (A: 0.1% formic acid, 2% acetonitrile in water and B: 0.1% formic acid in 80% acetonitrile) at 300 nl/min was used to elute peptides from the capillary reverse-phase column (0.075 mm by 250 mm, Pepmap, Dionex). Data were acquired using the Xcalibur software (version 4.2). A cycle of one full-scan mass spectrum [350 to 1500 mass/charge ratio (*m/z*)] at a resolution of 120,000 (at 200 *m/z*), followed by 20 data-dependent tandem mass spectrometry (MS/MS) spectra (at a resolution of 30,000; isolation window, 1.6 *m/z*) was repeated continuously throughout the nano-LC separation. Raw data analysis was performed using the MaxQuant software (version 1.6.10.43) with standard settings. Used database consist of *Homo sapiens* Reference proteome entries from Uniprot (www.uniprot.org, V2021_04, UP000005640) and 250 classical contaminants (MaxQuant contaminant database). Enzyme specificity was set to trypsin/P, and the search included cysteine carbamidomethylation as a fixed modification and oxidation of methionine, and acetylation (protein N terminus) as variable modifications. Up to two missed cleavages were allowed for protease digestion. False discovery rate was set at 0.01 for peptides and proteins and the minimal peptide length at 7.

### Mass spectrometry analysis of truncated tubulin C-terminal peptides

Sample were prepared and analyzed as previously described (*23*). Briefly, after separation by SDS–polyacrylamide gel electrophoresis (SDS-PAGE), α- and β-tubulin regions were isolated and proteins were in-gel digested using trypsin (1 μg per band; Gold; Promega) for α-tubulin or AspN (1 μg per band; Pierce) for β-tubulin. Peptides were then analyzed online by nanoflow HPLC–nanoelectrospray ionization using a Q Exactive HF mass spectrometer (Thermo Fisher Scientific) coupled to a nano-LC system (U3000-RSLC, Thermo Fisher Scientific). A gradient consisting of 0 to 40% B in A for 60 min (A: 0.1% formic acid, 2% acetonitrile in water and B: 0.1% formic acid in 80% acetonitrile) at 300 nl/min was used to elute peptides from the capillary reverse-phase column (0.075 mm by 250 mm, Pepmap, Dionex). Data were acquired using the Xcalibur software (version 4.0). A cycle of one full-scan mass spectrum (375 to 1500 *m/z*) at a resolution of 60,000 (at 200 *m/z*), followed by 12 data-dependent MS/MS spectra (at a resolution of 30,000; isolation window, 1.2 *m/z*) was repeated continuously throughout the nano-LC separation. Raw data analysis was performed using the MaxQuant software (version 2.0.3.0) with standard settings. Used database consist of *H. sapiens* Reference proteome entries from Uniprot (www.uniprot.org, V2022_01, UP000005640), and tubulin sequences (Q71036, P07437 and P68371) deleted from one to five amino acids in C terminus and 250 classical contaminants (MaxQuant contaminant database). Relative abundance of peptide was estimated using Skyline 20.1.

### In silico search for metalloprotease candidates

We first established a list of proteins that were specifically enriched in fraction 15 compared to fraction 11 using a fourfold enrichment cutoff based on spectral count (MS/MS) ratios. This resulted in a short list of 409 candidates that are summarized in table S1. To narrow down the putative metalloenzymes from this list, a computational analysis using the online ScanProsite tool (Expasy) was performed by submitting different metal-binding motifs (HEXXH, HEXXXH, and HXXEH) and scanning them against the preselected candidates. A total of 11 proteins containing the HEXXH motif, 11 proteins containing the HEXXXH motif, and 11 proteins containing the HXXEH motif were identified (highlighted in table S1).

### Anti–βIΔ3-tubulin antibody production

Anti–βI∆3-tubulin antibody was raised in rabbit by Eurogentec using a Speedy 28-day program against the peptide -DFGEEAE and affinity purified against the same peptide.

### Western blot analysis

SDS-PAGE was performed as previously described to separate α- and β-tubulin (*5*). Proteins were transferred onto 0.45-μm nitrocellulose membranes (Amersham), blocked with 5% milk in tris-buffered saline–Tween 20 followed by immunodetection. Membranes were incubated with rabbit polyE (anti-polyglutamylation; 1:1000), mouse anti-GFP (1:5000; Torrey Pines Biolabs), rabbit anti-GST (1:2000), rabbit anti–detyrosinated α-tubulin [1:1000; (*39*)], rabbit anti–Δ2-tubulin (1:1000; gift of L. Lafanechère), mouse 6-11B-1 (anti–acetylated tubulin, 1:2000; Sigma-Aldrich), mouse 12G10 (anti–α-tubulin; 1:1000, DSHB), rat anti–tyrosinated tubulin [YL1/2; 1:1000; (*55*)], rabbit anti–βI-Δ3 (1:1000; this study), mouse anti-βI-tubulin (1:1000; Sigma-Aldrich, SAB4200732), rabbit anti–Sf9-Δ1-α-tubulin [1:1000; (*23*)], mouse GT335 [1:1000; (*17*)] rabbit anti–hemagglutinin (HA) (1:1000; Santa Cruz Biotechnology) or mouse anti-His (1:1000; ProteinTech) antibodies. Protein bands were visualized with horseradish peroxidase–labeled goat anti-rabbit (Cell Signaling Technology), anti-mouse (Cell Signaling Technology), or anti-rat immunoglobulin G (Merck) all at 1:5000 followed by detection with chemiluminescence (SuperSignal West Pico PLUS, Thermo Fisher Scientific) using a Chemidoc Touch imaging system (Bio-Rad) or chemiluminescence films (GE Healthcare). For Fig. 3F, immunoblots were quantified using Image Lab software (Bio-Rad).

### Immunofluorescence

RPE1 cells were transfected with GFP-TMCP1 or GFP-TMCP2 isoforms as well as with His–α-tubulin and Strep–β-tubulin constructs using jetPEI (Polyplus) according to manufacturer’s protocol for 24 hours. Cilia induction in RPE1 cells was performed by serum starvation for 48 hours. Untransfected RPE1 cells, SH-SY5Y cells, and U2OS cells as well as transfected RPE1 cells were fixed with cold methanol for 5 min at −20°C. Immunostainings in mammalian cells were performed in phosphate-buffered saline (PBS) supplemented with 3% BSA and 0.1% Triton X-100. Primary antibodies used: anti–α-tubulin C102 (1:1000) (*56*), anti–∆1-tubulin (1:1000) (*39*), anti–∆2-tubulin (BioLegend, 909503) (1:1000), YL1/2 [anti–Tyr–α-tubulin; (*55*)] (1:1000), anti–acetylated α-tubulin (Sigma-Aldrich, T7451) (1:1000), anti–β-tubulin (E7, DSHB) (1:100), anti–βI-tubulin (Sigma-Aldrich, SAB4200732) (1:500), anti–βI∆3-tubulin (this paper) (1:500), anti-centrin (Sigma-Aldrich, 04-1624) (1:1000), and anti-StrepTag (GenScript, A01732) (1:500). Secondary antibodies coupled to Alexa Fluor 555 or Alexa Fluor 647 from Invitrogen Molecular Probes were used at 1:1000 and 1:600, respectively. Optical sections of the cells were acquired with a 40X Plan Apochromat 1.4–numerical aperture (NA) oil differential interference contrast (DIC) objective on a Zeiss Axioimager equipped with Apotome technology and an ORCA-Flash4 LT Hamamatsu monochrome camera to image the disappearance of YL1/2 and βI-tubulin staining upon overexpression of TMCP1 and TMCP2, respectively. All other acquisitions were performed with a 63X Plan Apochromat 1.4-NA oil DIC objective on a Leica SP8 confocal microscope equipped with hybrid detectors with a GaAsP photocathode.

### Expression constructs

TMCP1 open reading frame (ORF) was amplified from human SH-SY5 cell cDNAs with the primers 5′-CGCCGCGGATCCATGGTGCTGGACTCAGGG-3′ and 5′-CGCCGCAAGCTTTCAGTCGGGTAGCAGGCG-3′. The PCR product was digested with Bam HI and Hind III and cloned into a pEGFP-C1 vector (sites Bgl II and Hind III) to produce GFP-TMCP1, or cloned into a HA-pRK5 vector (sites Bam HI and Hind III) to produce HA-TMCP1, or cloned into pET28A+ (sites Bam HI and Hind III) to produce His-TMCP1. Point mutations in TMCP1 were introduced with a Q5 Site-Directed Mutagenesis Kit (NEB, E0552S) using the primers 5′-ATGCCGCAGCATGCCCTC-3′ and 5′-GCGATAGGCACCCACTACCTG-3′ for E281A mutation and primers 5′-CTCCGTGGGGTTCGCCGG-3′ and 5′-GCGGGCCTGGCCAGCCTG-3′ for E316A mutation.

*TMCP2* isoforms were amplified from human cDNAs using the reverse primer 5′-CGCGGAGAGCTCCTATATAAGATCTTTCAGTTCCCTGTC-3′ and isoform-specific forward primers: 5′-GCCAGCGGCCGCGGATGGCTGGCTGCACCCGC-3′ for *TMCP2-1*, 5′-CGCGGAGAGCTCATGCTGGAGTCCATTCGC-3′ for *TMCP2-2* and *TMCP2-3*, and 5′-CGCGGAGAGCTCATGAAAGCCCTGGTGCCT-3′ for *TMCP2-5*. All isoforms were cloned into a pcDNA3 vector containing N-terminal enhanced cyan fluoresent protein (eCFP) using Not I and Sac I sites. To produce His-tagged TMCP2-5, ORF was amplified with 5′-GCCAGCGGCCGCGGATGAAAGCCCTGGTGCCT-3′ and 5′-GCCAGCGGCCGCCTATATAAGATCTTTCAGTTCCCTGTC-3′ and cloned into the Not I site of pET28A. Point mutations in TMCP2-3 isoform were introduced with the Q5 Site-Directed Mutagenesis Kit using the primers 5′-ATGCCTCAGCATTCCCTCCA-3′ and 5′-GCGATAGGTACACATTATTTTCGAGGTATT-3′ for E329A mutation and primers 5′-CTCTGTGGGATTATTTGGCTTTAG-3′ and 5′-GCGGGACTAGCAAGCATTCACAGT-3′ for E364A mutation (numbers refer to *TMCP2-3* isoform sequence).

A codon-optimized version of *Tetrahymena thermophila TMCP* was synthetized by IDT with the sequence: 5′-ATGGATAAGAAAAAAATCTCTACGAATAAGAACTCTCAACTTCAAACTCCCAAGGAGGCCAAAACCGTAACAAAAAAAGCTACTATTCGAACCCAAAGCGTGTCTCCTCAGATTAAATTGTTTGCTAAAGAAAGCATAATAAATACCTACGCCGCGCTCGCAACACCTTCACCACAAAAGAGTCTTAACCAGAAGGAGCTCGCAAAAATTAAGAAAAATGTAGTCAACCAGCTGAAGAACAAATCTTCTCTGATGGCTTCTGGGATAGCGGTTGTATTGCAAAAACCGGTGCAAAAACTTTCCTCCAACAATCTCTCCTCCGCAAGCAGCAAAGCTGACTGTCTCATCGACAACGGAAAAAATATGAAAAATTTGTCCAGCTTTACGGAAGTGAATGTTTGCCTTAACACCCAAAATAAGGAAAATAACTGTCAACAGAACGCCACAAAAGACAAAAAGGCAAAGAAAGATAAAAAGACGGCCCTCAAAGACCTGAAGCCGCTGAATCTTGAGAAAGAGAAAGAGCTTTTCTTTAAATCTAATTTCACTTACAACCCCAACTTTCTGTACCAATTGTCTGATATCAACCTGAACTATTCAGTCCCTCACACAAAGTATTTCGACTTGGCTCTGAAAATATTGAACGAGGTTATTAAAATATATGGTAGTGAGCACAAGTTCAATGAAGAAGAAGGCGGCCAAATCATTGATATCCCGACTACTACGTCCTTTTTCGAGAAATACGTTAAGGATTTGGAGTGTGAGGACCTCGTAACATACGAATTTCAAAGCAACACCATTAGCCCTACTTGCTGCCAACACAACAAAGATGGCACAAGTAAAATCATAATAGGTCTGCCCATCCTTTATCGAGAACTTCGAATCCAAGGCGTCCTCAACCATGAAATTGGTACTCACTTCCTTCGCAAGCATAACGAGCGGCAGCAGGTATGGTTCAAGCAAAGGAAGAAATTTGGACTCGAACCCTCCTCTAAGGTTGAGGAGGGCCTCGCCCTGGTCAACCAGAAATACGATTTGGCTTTGAATCAAAGCCAGAAGCCCTTCGTCTTCAATGCCGCTCTTCACTATTATGCGTCAGTTAAGAGCAGCCAGATGAGCTTTAGCGAATTGTTTAATGACCTTGAAAAGTACGTGAAGGACCCAGACAAACGCTGGCGAGAATGCTTGAGGGTGAAGCGAGGTATTTCTGATACTTCCCAGCACCTGGGAATGTATAAAGATCAGATCTACTTGATTGGGATTAGACGCGTCCTGAAGTATAGGAACAAGATTGATCTCGTAAAGCTCCACTCTGGGAAAATCACCGTCAAGGACTGCATTAAGCTCACAGAGAAAAATCTTATTAATACTGAGAAGATCAAGATACCGTATTTTCTGAAGGATATTAATTTGTATAAAAAGGCACTCGATCGCATCGCCGAAGTAAACTTTATGTCAGATATTACAGTTCTTTGA-3′. The TtTMCP sequence was amplified by PCR with the primers 5′-CGCCGCGGATCCATGGATAAGAAAAAAATCTCTACG-3′ and 5′-CGCCGCAAGCTTTCAAAGAACTGTAATATCTGACAT-3′ and cloned by restriction into Bam HI and Hind III sites of HA-pRK5 vector. To produce a dead version of this construct (TtTMCP-E302A), a point mutation was inserted with the NEB Q5 Site-Directed Mutagenesis Kit using the primers: 5′-ATGGTTGAGGACGCCTTGGA-3′ and 5′-GCAATTGGTACTCACTTCCTTCGCA-3′.

For α-tubulin clones, a cDNA corresponding to the isotype *TubA1B* was amplified from human SH-SY5 cell cDNAs using the primers 5′-CGGACTCGAGACCATGCGTGAGTGCATCTCCATCC-3′ and 5′-CGCCGCGGATCCTTAGTATTCCTCTCCTTCTTCC-3′, digested with Xho I and Bam HI, and cloned into Xho I and Bam HI sites of pcDNA3.1(−) vector. Then, a 6xHis-tag was inserted after residue 43 in the TubA1B sequence with the Q5 Site-Directed Mutagenesis Kit using the primers 5′-CACCACCATGGAGGAGATGACTCCTTCAA-3′ and 5′-GTGATGGTGCCCAATGGTCTTGTCACTTG-3′. The 6xHis-tag at this position is inserted in a luminal loop of α-tubulin, and it was previously shown that this insertion does not affect polymerization of yeast tubulin (*24*). This His-TubA1B clone was used as a template to generate mutations of the C-terminal tails using the Q5 Site-Directed Mutagenesis Kit using the primer pairs indicated in Table 1.

**Table 1.** Sequences of primers used to mutagenize α-tubulin clones.

Tail sequence	Forward primer (5′-3′)	Reverse primer (5′-3′)
VEGEGEEEGEE	TCCTTCTTCCTCACCCTCTCCTTCAA	GAAGAGTAAGGATCCGAGCTCGGTACC
VEGEGEEEGE	TCCTTCTTCCTCACCCTCTCCTTCAA	GAGTAAGGATCCGAGCTCGGTACC
VEGEGEEEGEEEY	TCCTTCTTCCTCACCCTCTCCTTCAA	GAAGAGGAATACTAAGGATCCGAGCTCGGTACC
VEGEGEEEGEEE	TCCTTCTTCCTCACCCTCTCCTTCAA	GAAGAAGAGTAAGGATCCGAGCTCGGTACC
VEGEGEEEGEDY	TCCTTCTTCCTCACCCTCTCCTTCAA	GAGGATTACTAAGGATCCGAGCTC
VEGEGEEEGED	TCCTTCTTCCTCACCCTCTCCTTCAA	GAAGATTAAGGATCCGAGCTCGGTACC
VEGEGEEEGEEF	TCCTTCTTCCTCACCCTCTCCTTCAA	GAGGAATTCTAAGGATCCGAGCTCGGTACC
VEGEGEEEGEQY	TCCTTCTTCCTCACCCTCTCCTTCAA	GAGCAGTACTAAGGATCCGAGCTCGGTACCA
VEGEGEEEGEQ	TCCTTCTTCCTCACCCTCTCCTTCAA	GAGCAGTAAGGATCCGAGCTCGGTACCA
VEGEGEEEGEEA	TCCTTCTTCCTCACCCTCTCCTTCAA	GAGGAAGCTTAAGGATCCGAGCTCGGTACC
VEGEGEEEEEEY	TTCTTCTTCCTCACCCTCTCCTTCAA	GAGGAATACTAAGGATCCGAGC
VEGEGEEEEEE	TTCTTCTTCCTCACCCTCTCCTTCAA	GAAGAGTAAGGATCCGAGCTCGGTACC
VDGDGDDDGE (used as template for downstream clones)	TCCATCGTCGTCACCATCTCCATCAACAGAATCCACACCAACCTC	GAGTAAGGATCCGAGCTCGGTACC
VDGDGDDEGE	TTCGTCGTCACCATCTCCATCAA	GGAGAGTAAGGATCCGAGCT
VDGDGDEEGE	TTCTTCGTCACCATCTCCATCAA	GGAGAGTAAGGATCCGAGCT
VDGDGEEEGE	TTCTTCCTCACCATCTCCATCAA	GGAGAGTAAGGATCCGAGCT
VDGDGGEEGE	TTCTTCCCCACCATCTCCATCAA	GGAGAGTAAGGATCCGAGCT

To produce GFP-tagged variants of α-tubulin, enhanced GFP (eGFP) was inserted by recombination into the His-αTub1B vectors containing the appropriate tail mutations. eGFP was amplified with the primers 5′-GCGTTTAAACGGGCCCTCTAGAATGGTGAGCAAGGGCGAGGA-3′ and 5′-GATGCACTCACGCATGGTCTCGAGCTTGTACAGCTCGTCCATGC-3′ and inserted using the NEBuilder HiFi DNA Assembly (NEB E2621S) into the His-αTub1B vectors opened with Xho I.

To produce GST–His–α-tubulin variants, the His-TubA1B sequences carrying various mutant tails were amplified by PCR using the primers 5′-CCGCAGATCTATGCGTGAGTGCATCTCCATC-3′ and 5′-TTACTCGAGGTACCGAGCTCGGATCCTTA-3′, then digested with Bgl II and Xho I, and cloned into Bam HI and Xho I sites of pGEX6.1 vector.

For β-tubulin clones, three isotypes (*TubB1*, *TubB2A*, and *TubB4B*) were amplified from human SH-SY5 cell cDNAs and cloned into the Eco RI and Xho I sites of pGEX6.1 vector. Primers used were as follows: 5′-CGCGAATTCATGAGGGAAATCGTGCACATC-3′ and 5′-CGGACTCGAGTTAGGCCTCCTCTTCGGC-3′ for *TubB1*, 5′-CGCGAATTCATGCGCGAGATCGTGCAC-3′ and 5′-CGGACTCGAGTTAAGCCTCGTCCTCGCC-3′ for *TubB2A*, and 5′-CGCGAATTCATGCGGGAGATCGTGCAC-3′ and 5′-CGGACTCGAGCTAGGCCACCTCCTCCTC-3′ for *TubB4B*. Mutations of the C-terminal tails of each β-tubulin isotype were introduced by PCR using the WT clone as the template and the primers indicated in the Table 2. All variants are cloned into the Eco RI and Xho I sites of pGEX6.1 vector.

**Table 2.** Sequences of primers used to mutagenize β-tubulin clones.

Isotype and tail sequence	Forward primer (5′-3′)	Reverse primer (5′-3′)
TubB1–GEEAEEE	CGCGAATTCATGAGGGAAATCGTGCACATC	CGGACTCGAGTTACTCCTCTTCGGCCTC
TubB1–GEEAEE	CGCGAATTCATGAGGGAAATCGTGCACATC	CGGACTCGAGTTACTCTTCGGCCTCCTCACC
TubB1–GEEAE	CGCGAATTCATGAGGGAAATCGTGCACATC	CGGACTCGAGTTATTCGGCCTCCTCACCGAA
TubB1–GEEA	CGCGAATTCATGAGGGAAATCGTGCACATC	CGGACTCGAGTTAGGCCTCCTCACCGAAATC
TubB2A–GEDEA	CGCGAATTCATGCGCGAGATCGTGCAC	CGGACTCGAGTTAAGCCTCGTCCTCGCC
TubB2A–GEEEA	CGCGAATTCATGCGCGAGATCGTGCAC	CGGACTCGAGTTACTCGCCCTCCTCCTC
TubB4B–AEEEV	CGCGAATTCATGCGGGAGATCGTGCAC	CGGACTCGAGCTACACCTCCTCCTCCGC
TubB4B–AEEE	CGCGAATTCATGCGGGAGATCGTGCAC	CGGACTCGAGCTACTCCTCCTCCGCCTC
TubB4B–AEE	CGCGAATTCATGCGGGAGATCGTGCAC	CGGACTCGAGCTACTCCTCCGCCTCCTC
TubB4B–AE	CGCGAATTCATGCGGGAGATCGTGCAC	CGGACTCGAGCTACTCCGCCTCCTCCTC

To produce Strep-tagged β-tubulins, the Strep-tagII sequence was inserted in βI- or βIV-tubulin sequences after amino acid 34 using a Q5 mutagenesis kit (Biolabs) and the primers 5′-CAGTTCGAAAAGACCUACCACGGGGACAGC-3′ and 5′-CGGGTGGCTCCAGCCCGTGGGGTCGATGCC-3′.

A bicistronic *VASH2-T2A-SVBP* sequence was synthetized by IDT and cloned into Xho I and Xba I sites of pCDNA3.1(+) vector. The codon-optimized sequence is 5′-CTCGAGCACCATGACAGGTAGTGCGGCAGATACTCATCGCTGCCCACACCCGAAAGGTGCAAAGGGTACTCGCAGCCGCAGTAGTCACGCGCGACCAGTGAGTTTGGCGACTTCGGGAGGTAGCGAGGAGGAGGATAAAGATGGTGGTGTTCTGTTCCATGTAAACAAATCCGGATTCCCGATAGACTCCCACACATGGGAACGCATGTGGATGCATGTGGCAAAAGTGCACCCAAAGGGAGGTGAGATGGTCGGAGCTATACGTAATGCAGCTTTCCTGGCAAAACCCAGTATTCCACAAGTGCCCAATTATCGCTTGAGTATGACTATCCCCGACTGGCTCCAAGCAATACAAAATTACATGAAGACGCTCCAATACAACCACACTGGTACCCAGTTCTTCGAGATACGCAAGATGCGCCCGCTCTCCGGATTGATGGAGACGGCTAAGGAGATGACTCGTGAGTCGTTGCCCATCAAGTGTCTCGAAGCCGTGATCTTGGGCATTTATCTCACGAATGGACAACCAAGTATCGAGAGGTTTCCCATAAGTTTCAAGACCTATTTTTCGGGCAACTACTTCCACCACGTAGTCCTGGGAATCTACTGTAATGGCAGGTACGGTTCCCTCGGTATGAGTCGCCGCGCGGAGCTGATGGACAAACCTCTGACTTTTCGGACGTTGAGCGATTTGATTTTTGACTTTGAAGATAGCTATAAGAAATATTTGCACACTGTTAAGAAAGTGAAAATCGGTTTGTACGTCCCGCATGAACCCCACTCGTTTCAGCCTATCGAGTGGAAGCAGCTGGTGCTGAACGTCAGCAAGATGCTGAGGGCAGACATCCGCAAGGAGCTGGAGAAATACGCGAGGGACATGCGTATGAAAATTTTGAAACCAGCAAGCGCGCATTCCCCCACGCAGGTGCGTAGTAGGGGTAAGAGTCTGAGTCCTCGGCGTCGTCAGGCGTCGCCCCCACGTCGCTTGGGCAGGAGGGAGAAGAGCCCGGCGCTGCCCGAGAAGAAAGTCGCCGACCTGTCGACATTGAATGAGGTCGGTTACCAGATTCGAATCCGGGCGGAAGGACGGGGTTCCCTGCTCACATGCGGAGACGTTGAAGAGAACCCGGGTCCAATGGATCCCCCAGCGAGGAAGGAGAAAACAAAAGTTAAAGAATCGGTCTCCAGGGTGGAAAAAGCGAAGCAAAAGAGCGCACAGCAAGAGTTGAAACAAAGGCAGCGAGCGGAGATTTATGCGCTCAACCGCGTTATGACGGAGCTGGAGCAGCAGCAGTTCGACGAGTTTTGCAAGCAAATGCAACCACCAGGCGAGTGACTCTAGA-3′. To produce a dead version of this bicistronic construct, a point mutation was inserted with the NEB Q5 Site-Directed Mutagenesis Kit using the primers 5′-GCTCTCGAAGCCGTGATCTTGGG-3′ and 5′-CTTGATGGGCAACGACTCACG-3′ to produce *VASH2(C158A)-T2A-SVBP* construct.

For all PCRs, the Q5 High-Fidelity DNA Polymerase was used (NEB M0494L). All clones and point mutations listed above were verified by sequencing. Plasmids encoding TTLL6, TTLL11, and TTLL13 were previously described (*19*).

### Expression and purification of recombinant proteins

Transformed *Escherichia coli* strain BL21 Star (DE3) were grown at 37°C until the optical density at 600 nm reached 0.6, and then the culture was induced with 0.4 mM isopropyl-β-d-thiogalactoside overnight at 18°C. Cell pellets were resuspended in 50 mM tris-HCl (pH 7.5), 500 mM NaCl, and 1 mM Tris(2-carboxyethyl)phosphine (TCEP). Cell lysis was performed by sonication followed by centrifugation at 20,000*g* for 30 min at 4°C. The supernatant was incubated with prewashed Ni-NTA (nitrilotriacetic acid) agarose beads (Qiagen) or glutathione beads (GE Healthcare) at 4°C for 3 hours. Beads were washed extensively in lysis buffer supplemented with 20 mM imidazole and 0.1% Triton X-100 (His-tag purification) or lysis buffer with 0.1% Triton X-100(GST-tag purification). Elutions were performed in either 50 mM tris-HCl (pH 7.5), 500 mM NaCl, 250 mM imidazole, and 1 mM TCEP for His-tag purification or in 50 mM tris-HCl (pH 7.5), 500 mM NaCl, 20 mM reduced glutathione, and 1 mM TCEP for GST-tag purification. The elution fractions containing proteins were dialyzed overnight at 4°C against 50 mM tris-HCl (pH 7.5), 300 mM NaCl, and 1 mM TCEP using 10-kDa dialysis cassettes. The dialyzed samples were then concentrated and buffer exchanged in 50 mM tris-HCl (pH 7.5), 200 mM NaCl, and 1 mM TCEP using 10-kDa Amicon centrifugal filter units (Amicon Ultra-4 Merck Millipore). For the purification of His-TMCP1 and His-TMCP2, a final polishing step using Superdex200 gel filtration column was performed. All recombinant proteins were snap frozen and stored at −80°C in aliquots.

### In vitro enzymatic assays

Enzymatic reactions were performed by incubating purified recombinant enzyme (50 nM final) with 2 μg of purified GST-tubulin or Sf9 tubulin or MTs in a buffer containing 50 mM tris-HCl (pH 7.5) and 10% glycerol (50 μl final volume) and incubated at 37°C for 2 hours by default or in time-course experiments. Samples were then mixed with loading buffer, boiled for 5 min at 95°C, and subjected to immunoblotting. To prepare MTs, tubulin was purified from Sf9 cells or mouse brain as previously described (*23*), resuspended in 80 mM Pipes (pH 6.8) and 10% glycerol, and incubated at 37°C for 30 min in the presence of 1 mM GTP and 20 μM paclitaxel. After polymerization, MTs were pelleted by ultracentrifugation at 100,000*g* for 30 min at 37°C, resuspended in 80 mM Pipes (pH 6.8) and 10% glycerol with 20 μM paclitaxel, and snap frozen and stored at −80°C in aliquots.

### Tetrahymena

The *TMCP* KO *Tetrahymena* strains were produced by codeletion (coDel) as described in (*57*). The target sequence for coDel was amplified from genomic DNA of the WT B2086 strain by PCR with TMCP_coDel_FW and TMCP_coDel_RV. Complete removal of the target TMCP coding region was confirmed by direct cell PCR with TMCP_coDel_cFW and TMCP_coDel_cRV. Primers used were as follows: TMCP_coDel_FW: 5′- CTTTATTGTTATCATCTTATGACCGCTTCTCCAACATGCTGCTAGC-3′; TMCP_coDel_RV:5′-CTCATCAAGTTGTAATGCTAAAATGCAAAGCACAGTTATATCGCTC-3′; TMCP_coDel_cFW: 5′-TATGCTGCATTAGCTACACC-3′; TMCP_coDel_cRV: 5′-AAAGTGATTAATTTATACTTGAGATCC-3′. Expression of *TtTMCP* was analyzed by RT-PCR using the primers 5′-TGACATTCCCACAACAACATCC-3′ and 5′-TGCTTTCTGAGAAAATGTGTTCCT-3′. The reference *L21* ribosomal gene was amplified using the primers 5′- AAGTTGGTTATCAACTGTTGCGTT-3′ and 5′- GGGTCTTTCAAGGACGACGTA-3′.

For growth curve experiments, WT B2086 and *TMCP* KO strains were cultured in Super Proteose Peptone (SPP) medium (*58*) at 30°C overnight. A total of 7.5 × 10^5^ cells were inoculated into 25 ml of fresh SPP medium in a 125-ml flask (3 × 10^4^ cells/ml) and incubated at 30°C with shaking at 90 rpm. Growth was monitored by counting cells using a model ZB1 Coulter counter (Coulter Electronics Inc., Hialeah, FL).

To overexpress the GFP- or HA- N-terminally tagged TtTMCP protein, the 2.2-kb *TMCP* coding sequence was amplified by PCR from the WT genomic DNA (CU428 strain) using Phusion Hot Start II high-fidelity DNA polymerase (Thermo Fisher Scientific Baltics, Lithuania) and the following primers: TtTMCP-MluI-F [5′-AATTACGCGTTATGGATAAGAAGAAGATCTCTACAAATAAAAATAGTTAGTTGT AAACG-3′ with two silent mutations (in italic)] and TtTMCP-Bam HI-R (5′-AATTGGATCCTCAAAGCACAGTTATATCGCTC-3′) and cloned into pNeo5-MTT1-GFP-BTU1 plasmid containing neo5-MTT1-GFP fragment (*59*), enabling the overexpression in nonessential *BTU1* locus. To obtain TMCP with E302Q mutation, two fragments of the *TMCP* coding region were amplified. The 1.4-kb upstream fragment was amplified using a TtTMCP-MluI-F primer and a primer with an introduced mutation resulting in E302Q substitution and directly below, a silent mutation leading to Kpn I restriction site formation: TtTMCP-E302Q-KnpI-R (5′-AATTGGTACCTATCTAATGATTTAAAACTCCTTATATTCTAAGTTC-3′). The 0.8-kb downstream fragment was amplified using TtTMCP–Kpn I–F primer (5′-AATAGGTACCCATTTTCTCAGAAAGCACAATGA-3′) and TtTMCP–Bam HI–R. The DNA fragments were digested with appropriate restriction endonucleases (Mlu I and Knp I or Kpn I and Bam HI) and cloned into neo5-MTT1-GFP-BTU1 overexpression plasmid digested with Mlu I and Bam HI (three-pieces ligation method). Plasmids enabling overexpression of TMCP with N-terminal HA tag were obtained by cloning WT and mutated *TMCP* version into pNeo5-MTT1-HA-BTU1 plasmid, constructed by replacing of the *GFP* coding region by the *HA* coding region. Plasmids (pNeo5-MTT1-GFP-TtTMCP-BTU1, pNeo5-MTT1-GFP-TtTMCP-E302Q-BTU1, pNeo5-MTT1-HA-TtTMCP-BTU1, and pNeo5-MTT1-HA-TtTMCP-E302Q-BTU1) were digested with Apa I and Sac II restriction enzymes and introduced into WT CU428 *Tetrahymena* strain by biolistic transformation. Transformants were selected on SPP supplied with paromomycin (100 μg/ml) and grown on increasing concentration of a drug (up to 800 μg/ml of medium).

Before analyses, *Tetrahymena* cells carrying transgenes enabling protein overexpression were diluted to 10^5^ × cells/ml and grown for 4 hours in SPP medium supplied with cadmium chloride (2.5 μg/ml) to activate *MTT1* promoter and induce protein overexpression. To detect transgenic proteins, cells were fixed 1:1 with a mixture of 0.25% Triton X-100 and 4% paraformaldehyde (PFA) in a Pipes Hepes EGTA Magnesium (PHEM) buffer. The anti-HA and anti-GFP antibodies were used at the following final concentrations: monoclonal mouse anti-HA antibodies (Covance, Berkeley, CA, USA) (1:300), polyclonal rabbit anti-GFP antibodies (Abcam, Cambridge, UK) (1:6000), anti-centrin antibodies (EMD Millipore, Temecula, CA) at the 1:800 dilution.

For immunofluorescence analyses of the tubulin polyglutamylation, before cell fixation, WT cells were incubated for 10 min at 30°C in SPP medium containing India Ink. India Ink-containing dark food vacuoles were used as a WT cell’s marker in side-by-side cell analyses. WT and mutant cells (5 μl each) were mixed 1:1 on the same slide and immediately fixed with the same volume of 0.5% Triton X-100/PHEM and after 1 min fixed with 20 μl of 8% PFA/PHEM. After drying, samples were blocked for 10 min in 3% BSA/PBS and incubated overnight with a following primary antibodies: GT335 (1:2000) or polyE (1:2000). Images were recorded using a Leica TCS SP8 (Leica Microsystems, Wetzlar, Germany) confocal microscope.

For comparison of the level of tubulin glutamylation in WT and mutant cells, fluorescence intensity of 100 basal bodies was measured in five different *Tetrahymena* cells for each genotype using the ImageJ program. For comparison between WT and *TtTMCP* KO, fluorescence intensity of 50 basal bodies was measured in eight different *Tetrahymena* cells for each genotype. Average intensity value was arbitrary set to 100 for WTs. Statistical significances were addressed with nested *t* tests (GraphPad Prism).

For Western blot analyses, cytoskeletal cell extracts were prepared as described (*20*), and 2 μg of proteins obtained from WT cells overexpressing *Tt*TMCP normal and mutated variants and *TtTMCP* KO cells was loaded onto SDS-PAGE gel and analyzed by Western blot using following primary antibodies: GT335 diluted 1:4000, polyE diluted 1:20,000, anti-tubulin 12G10 diluted 1:80,000, anti-HA diluted 1:2000, and anti-GFP diluted 1:60,000.

### Phylogenetic tree

The tree of selected species was build using Timetree (*60*). The presence of TMCP homologs was determined using the EggNOG database (*61*).

### 3D protein structures

Overlay of the 3D structures were performed with USCF ChimeraX software. Structures used are 7Z5G [MATCAP/TMCP1; (*32*)], AF-Q7TQE7-F1 (mmTMCP2-3 isoform containing the alternative exon), and AF-Q8NCT3-F1 (hsTMCP2-1 isoform lacking the alternative exon). Note that hsTMCP2-1 isoform is identical to hsTMCP2-2 isoform, except in the N-terminal domain that is not represented in fig. S4.

### Droplet digital PCR

To quantify expression levels upon depletion by RNAi, we prepared cDNAs from cells transfected with various DsiRNA (see above) using the Maxima First-strand cDNA synthesis kit (Thermo Fisher Scientific). Expression levels were compared to the reference gene *TBP* by multiplex droplet digital PCR (ddPCR) using fluorescent probes sets. Primers and probes for each target gene were purchased from IDT. *TMCP2* primers: 5′-CGACTCTGACTATCAATGTGTCC-3′ and 5′-GGCTGCTGGAGGTTGTTAAT-3′. *TMCP2* probe: 5′-/56-FAM/TGGAGGGAA/ZEN/TGCTGAGGCATGAAA/3IABkFQ/-3′. *TMCP1* primers: 5′-GCGCAAATACATGCAGAAGG-3′ and 5′-TCAGGTTGATCGCCAATGT-3′. *TMCP1* probe: 5′-/56-FAM/AGTGATGAT/ZEN/GGTGGAGAACAGCCG/3IABkFQ/-3′. *TBP* mix of primers and probe: ref. #Hs.PT.58v.39858774. *CCP1* mix of primers and probe: ref. #HS.PT.58.3627499.

Multiplexed PCR were performed using ddPCR Supermix for Probes (Bio-Rad) and analyzed with a QX200 Droplet Digital PCR system (Bio-Rad).

### Purification of tubulin from HEK-2KO cells

Cell pellets were resuspended in one volume of 50 mM tris-HCl (pH 8), 50 mM KCl, 0.2% NP-40, and protease inhibitor cocktail (Roche). Cell lysis was completed by Dounce homogenization, and the suspension was rotated for 20 min before centrifugation at 15,000*g*. The clarified supernatant was further centrifuged at 100,000*g* for 30 min on a Beckman Optima TLX-120 Ultracentrifuge. The new supernatant was loaded onto a Mono Q 5/50 anion-exchange chromatography column (GE Healthcare) using a 500-μl sample loading loop on an Äkta purifier system (GE Healthcare). The column had been equilibrated with 50 mM tris-HCl (pH 8) as the binding buffer (buffer A). After sample injection and binding, the column was washed with 10 ml of buffer A. Bound proteins were then eluted over a linear gradient of buffer B (buffer A with 1 M KCl) with increasing ionic strength from 0 to 100% buffer B. Twenty fractions of 1 ml were collected at a flow rate of 0.5 ml min^−1^ for 40 min. The fractions containing tubulin (i.e., 11, 12, 13, and 14) were pooled together and concentrated and buffer exchanged in 80 mM Pipes buffer at (pH 6.8) with 10% glycerol using 10-kDa Amicon centrifugal filter units (Amicon Ultra-4 Merck Millipore).

### Purification of His-tag–α-tubulin and Strep-tag–β-tubulin dimers

HEK293 cells were transfected with plasmids coding for different His–α-tubulin variants or Strep–β-tubulin variants. After 48 hours of transfection, cells were collected and washed with PBS, and cell pellets were resuspended in lysis buffer containing 50 mM tris-HCl (pH 7.5), 200 mM NaCl, 0.2% NP-40, 1 mM TCEP, 0.5 mM EDTA, and anti-protease cocktail. Cell lysis was completed by Dounce homogenization and the suspension rotated at 4°C for 30 min. The suspension was centrifuged 20 min at 15,000*g* at 4°C, and the supernatant further ultracentrifuged at 100,000*g* at 4°C for 20 min. The supernatant was collected and added onto prewashed Ni-NTA agarose beads (His-tag purification) or Strep-Tactin 4Flow beads (Strep-tag purification). Binding was done overnight at 4°C with rotation. Beads were then washed extensively in lysis buffer and two times in tubulin buffer [80 mM Pipes (pH 6.8), 10% glycerol, and 1 mM TCEP] and kept at 4°C before enzymatic reactions.

### His–α-tubulin purification (denaturing conditions)

His-selectCobalt affinity gel (Sigma-Aldrich, USA #H8162) was used to purify histidine-tagged tubulins. Briefly, transfected HEK293T cells were washed twice with 4 ml of PBS and lysed at 4°C for 30 min with 0.65 ml of His-tag purification buffer [80 mM Pipes (pH 6.8), 0.2% NP-40, and 5.25 M urea]. The cell lysate was centrifuged at 21,000 rpm for 10 min. The protein supernatant (500 μl) was transferred to a 1.5-ml Eppendorf tube and mixed with 50 μl of prewashed cobalt beads. Tubes were rotated at 4°C for 3 hours and then centrifuged at 800 rpm for 2 min. The beads were washed three times with 1 ml of the wash buffer [80 mM Pipes (pH 6.8), 0.2% NP-40, and 5.25 M urea]. Following washing, bound proteins were released from the beads with 80 μl of Laemmli buffer and boiled at 95°C for 5 min before use.
