# Supplementary material for: A family of carboxypeptidases catalyzing α- and β-tubulin tail processing and deglutamylation
Source: Sci Adv. 2023 Sep 13;9(37):eadi7838. doi: 10.1126/sciadv.adi7838 (PMC10499314; doi:10.1126/sciadv.adi7838)
Supplement: Supplementary file 1 — Figs. S1 to S10 Legend for table S1 [file sciadv.adi7838_sm.pdf]

Science Advances
AAAS

Supplementary Materials for

A family of carboxypeptidases catalyzing α- and β-tubulin tail processing and deglutamylation

Simon Nicot *et al.*

Corresponding author: François Juge, francois.juge@inserm.fr;
Krzysztof Rogowski, krzysztof.rogowski@igh.cnrs.fr

Sci. Adv. **9**, eadi7838 (2023)
DOI: 10.1126/sciadv.adi7838

The PDF file includes:

Figs. S1 to S10
Legend for table S1

Other Supplementary Material for this manuscript includes the following:

Table S1

Supplementary Figure 1

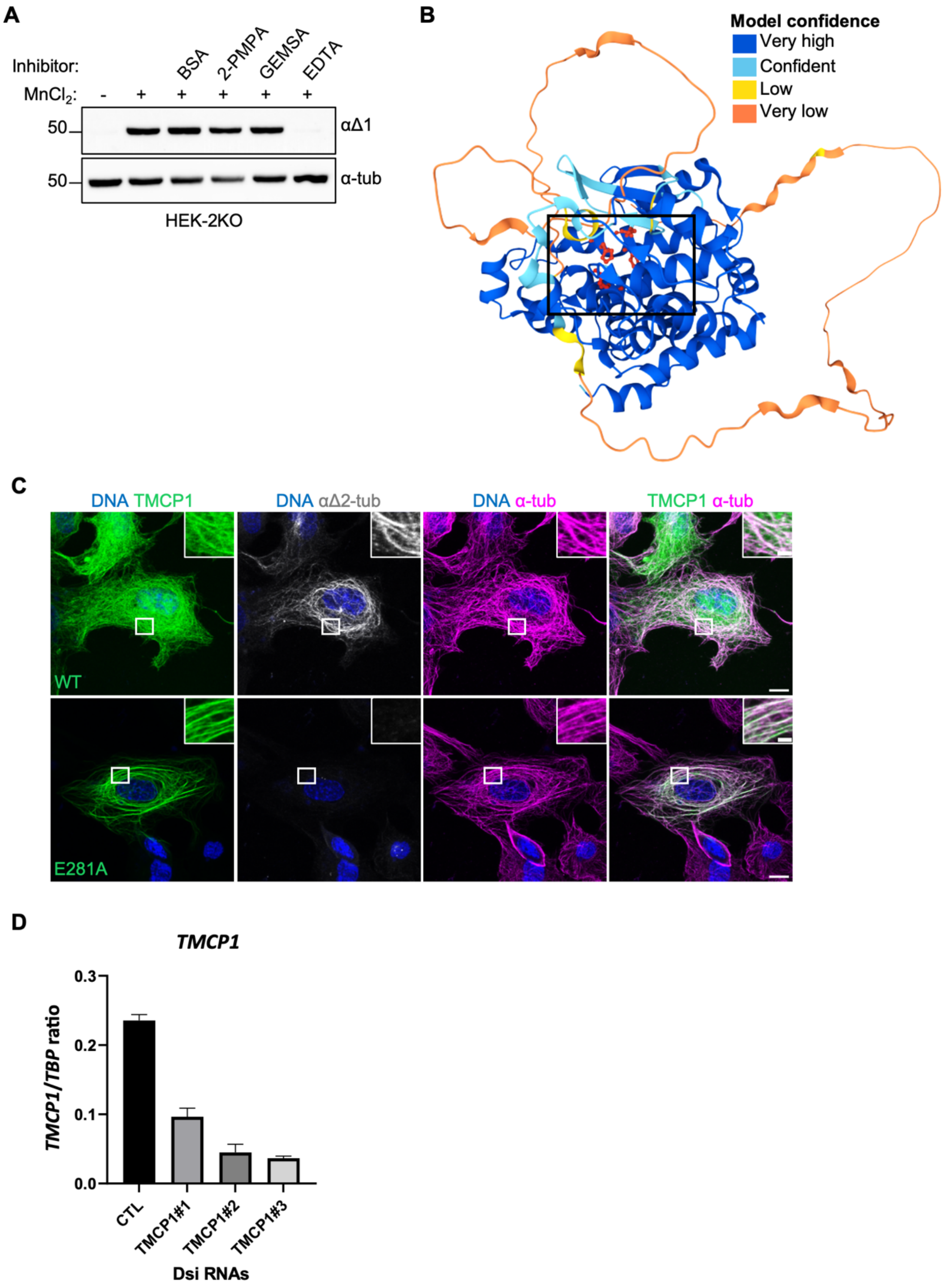

Fig. S1. Additional characterization of TMCP1. (**A**) Immunoblot analysis of *in vitro* detyrosination assay using extracts from HEK-2KO cells in the presence of various carboxypeptidase inhibitors including: Benzylsuccinic acid (BSA), 2-(phosphonomethyl)-pentanedioic acid (2-PMPA) and 2-guanidinoethylmercaptosuccinic acid (GEMSA). EDTA was used as a positive control. (**B**) AlphaFold generated prediction of KIAA0895L (TMCP1) structure. An enlarged view of the box centered around the active site is presented in Fig. 1F. (**C**) Immunofluorescence analysis of RPE1 cells expressing either the active or inactive (E281A) GFP-TMCP1. Note stronger MT association for the enzymatically inactive TMCP1. Scale bars: 10 µm, insets: 2 µm. (**D**) Graphical representation of knockdown efficiency of three different siRNAs targeting *TMCP1*. Transcripts levels were measured by digital droplet PCR following 48h knockdown in SH-SY5Y cells.

Supplementary Figure 2

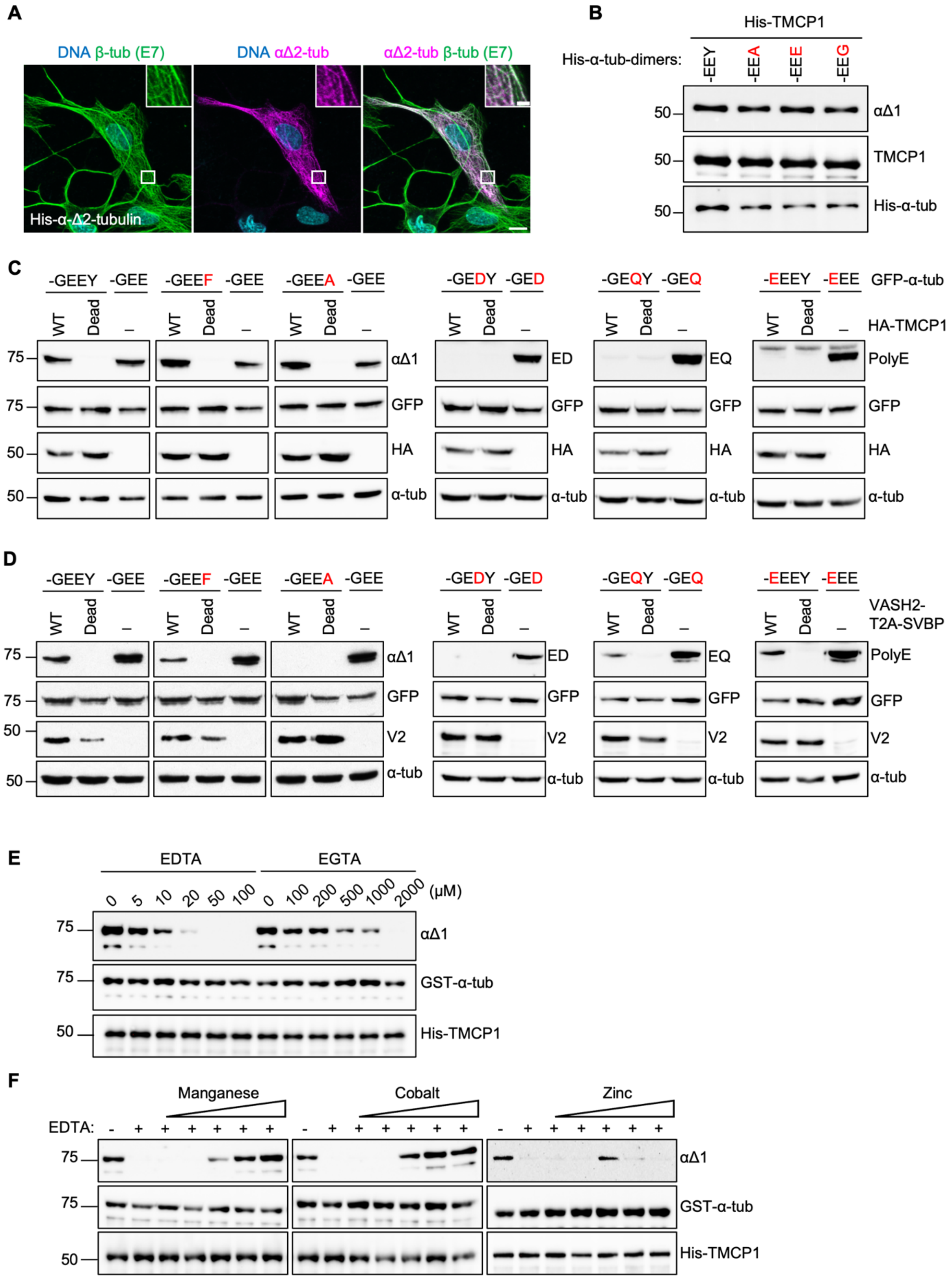

Fig. S2. Substrate specificity and metal preference of TMCP1. (**A**) Immunofluorescence analysis αΔ2-tubulin harboring an internal His-tag expressed in RPE1 cells. Insets show colocalization of αΔ2 signal with MTs. Scale bars: 10 µm, insets: 2 µm. (**B**) Immunoblot analysis of *in vitro* assays showing the specificity of TMCP1 towards tubulin dimers purified (His-tag purification) from HEK293T cells transfected with various His-α-tubulins carrying indicated amino acids at their C-terminus. TMCP1 generates αΔ1-tubulin modification regardless of the terminal residue identity on α-tubulin. (**C**) Immunoblots analysis of protein extracts from HEK-2KO cells co-expressing HA-TMCP1 or its enzymatically dead version (E281A) together with indicated GFP-α-tubulin mutants. GFP-α-tubulins mimicking modified variants were used as positive controls. (**D**) Immunoblot analysis of protein extracts from HEK-2KO cells co-expressing either active or inactive VASH2/SVBP together with indicated GFP-α-tubulin mutants. GFP-α-tubulins mimicking modified variants were used as positive controls. (**E**) Immunoblot analysis of *in vitro* activity assays involving recombinant TMCP1 and GST-α-tubulin in the presence of increasing concentrations of chelating agents EDTA and EGTA. Please note that although both chelators affected the activity of TMCP1, EDTA is a much more potent inhibitor in comparison to EGTA. (**F**) Immunoblot analysis of the metal replacement assay performed in the presence of EDTA. Mixtures of recombinant enzyme and substrate (His-TMCP1 and GST-α-tub) were pre-incubated with 50 µM EDTA (complete inhibition of the activity) for 10 minutes followed by addition of increasing amounts (10, 20, 50, 100, 200 µM) of divalent metal cations salts ($MnCl_2$, $CoCl_2$, $ZnCl_2$).

Supplementary Figure 3

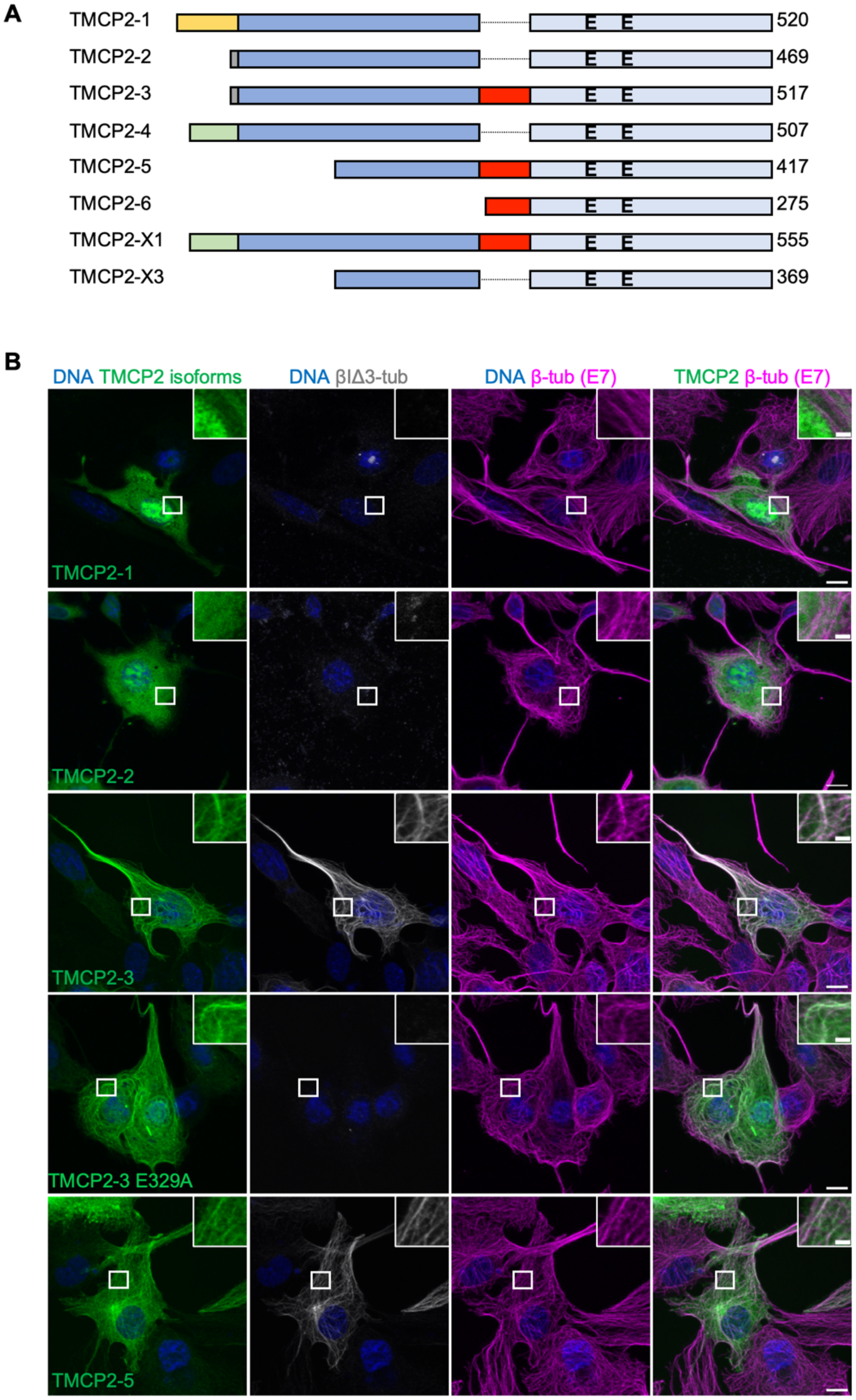

Fig. S3. Overview of TMCP2 isoforms and their subcellular localization. (A) Schematic representation of predicted human TMCP2 isoforms generated either by the usage of alternative promoters or alternative splicing (NCBI nomenclature). TMCP2-1 isoform is the only isoform harboring a nuclear localization signal (NLS) shown in yellow. The alternative exon number 5 (refers to the exon number downstream of the very first exon of the locus), associated with tubulin modifying activity, is highlighted in red. **(B)** Immunofluorescence analysis of RPE1cells expressing different GFP-TMCP2 isoforms. Whereas TMCP2-1 is localized in the nucleus, consistent with the presence of the NLS at the N-terminus, TMCP2-2, TMCP2-3 and TMCP2-5 show cytoplasmic localization. However, only TMCP2-3 and TMCP2-5, which harbor the alternative exon required for activity co-localized with MTs. Scale bars: 10 µm, insets: 2 µm.

Supplementary Figure 4

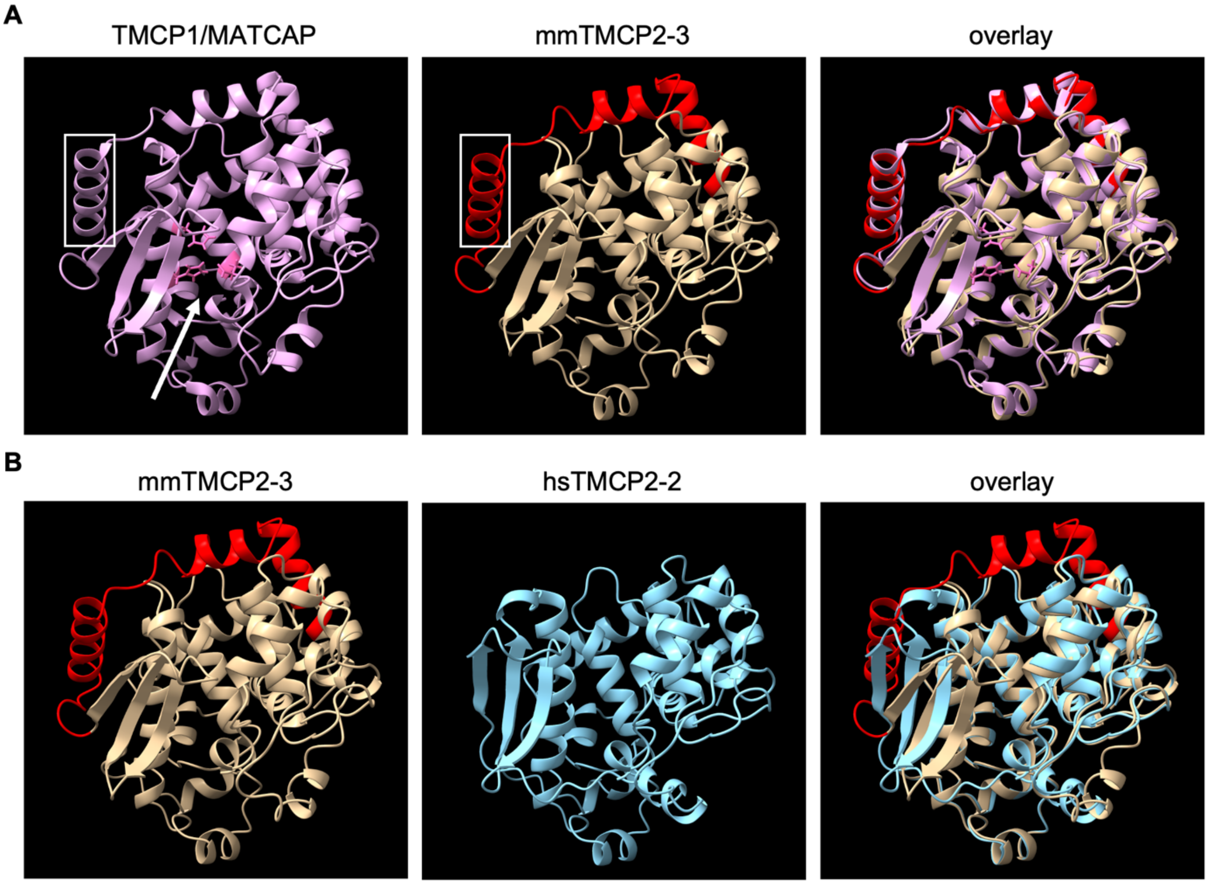

Fig. S4. Structural comparison of TMCP1 and TMCP2 isoforms. (**A**) The crystal structure of TMCP1/MATCAP is highly similar to the predicted structure of TMCP2 isoform containing the alternative exon. The alternative exon (highlighted in red) in TMCP2 includes a microtubule binding helix (framed) previously identified in TMCP1/MATCAP (*32*). The arrow indicates the location of the active site. (**B**) AlphaFold comparison of the predicted structures of TMCP2 isoforms containing or not the alternative exon. Absence of the alternative exon results in structural rearrangements, including a displacement of three beta sheets and movement of the sequences flanking the alternative exon in the front of the structure. In all images the N-terminal unstructured parts of the proteins are omitted.

Supplementary Figure 5

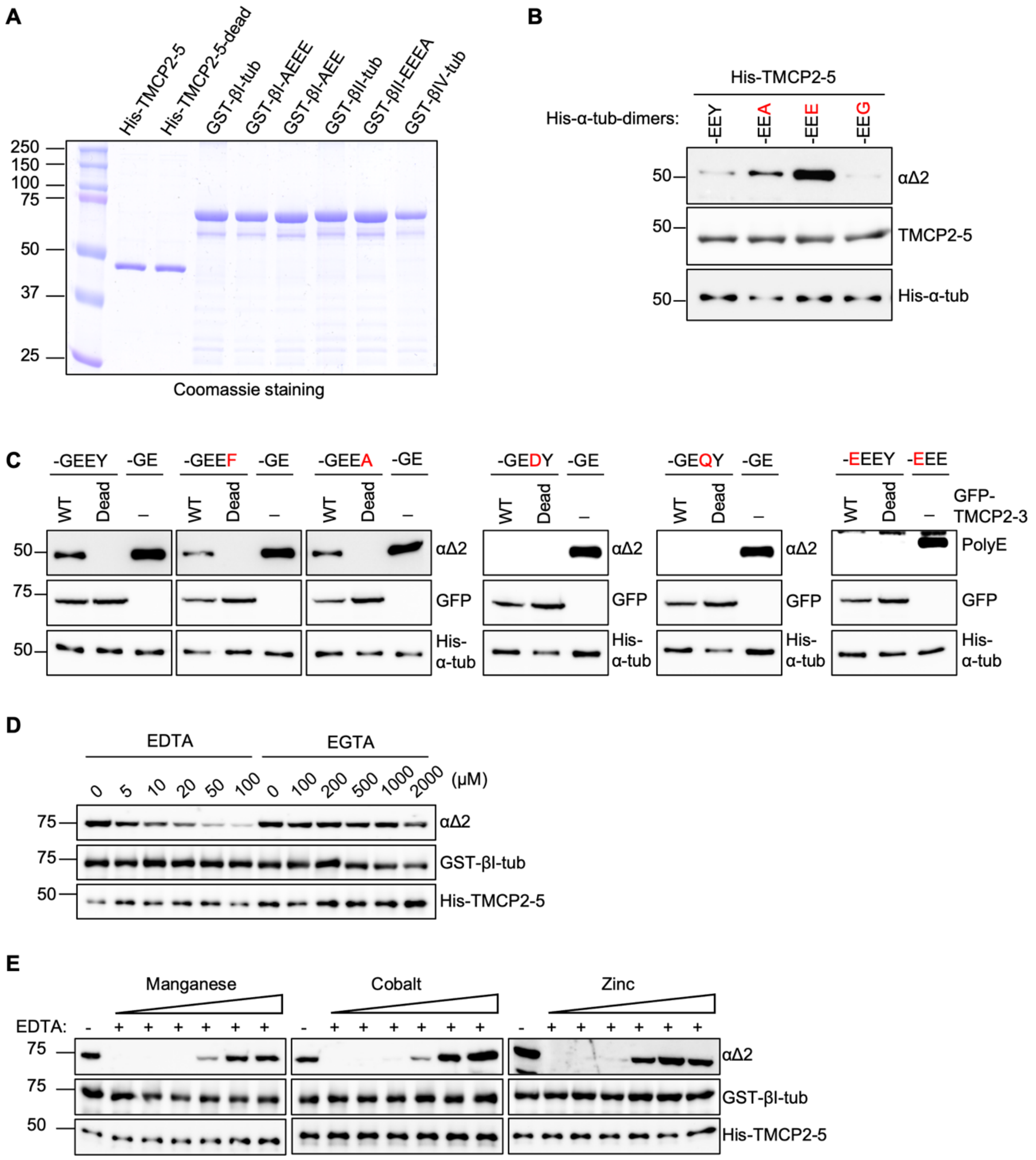

Fig. S5. Substrate and metal preference of TMCP2. (**A**) Coomassie stain gel of purified proteins used for *in vitro* studies of TMCP2. The following proteins were purified from bacteria: His-TMCP2-5, His-TMCP2-5-dead, GST-βI-tubulin and its truncated variants -AEEE and -AEE, GST-βII-tubulin, its mutated -EEEA variant and GST-βIV-tubulin. (**B**) Immunoblot analysis of *in vitro* specificity assays of TMCP2 towards tubulin dimers purified (His-tag purification) from HEK293T cells transfected with different His-α-tubulins constructs. TMCP2 is highly efficient at generating αΔ2-tubulin modification from α-tubulin ending with a glutamate, less efficiently with alanine or tyrosine while glycine is not modified. (**C**) Immunoblot analysis of HEK-2KO cells co-transfected with either active or enzymatically dead versions of GFP-TMCP2-3 and various His-α-tubulin isoforms carrying different tail modifications (modified residues are highlighted in red). His-α-tubulin lacking the last two residues (-GE) or lacking the C-terminal tyrosine (-EEE) were used as controls. (**D**) Immunoblot analysis of TMCP2-5 activity assays involving GST-βI-tubulin as a substrate in the presence of increasing concentrations of the chelating agents EDTA and EGTA. Please note that EDTA is a much more potent inhibitor as compared to EGTA. (**E**) Immunoblot analysis of the metal replacement assays in the presence of EDTA. Recombinant enzyme:substrate (TMCP2-5:GST-βI-tub) mixtures were pre-incubated with 50 µM EDTA for 10 minutes followed by addition of increasing amounts (10, 20, 50, 100, 200 µM) of metal divalent cation salts ($MnCl_2$, $CoCl_2$, $ZnCl_2$).

Supplementary Figure 6

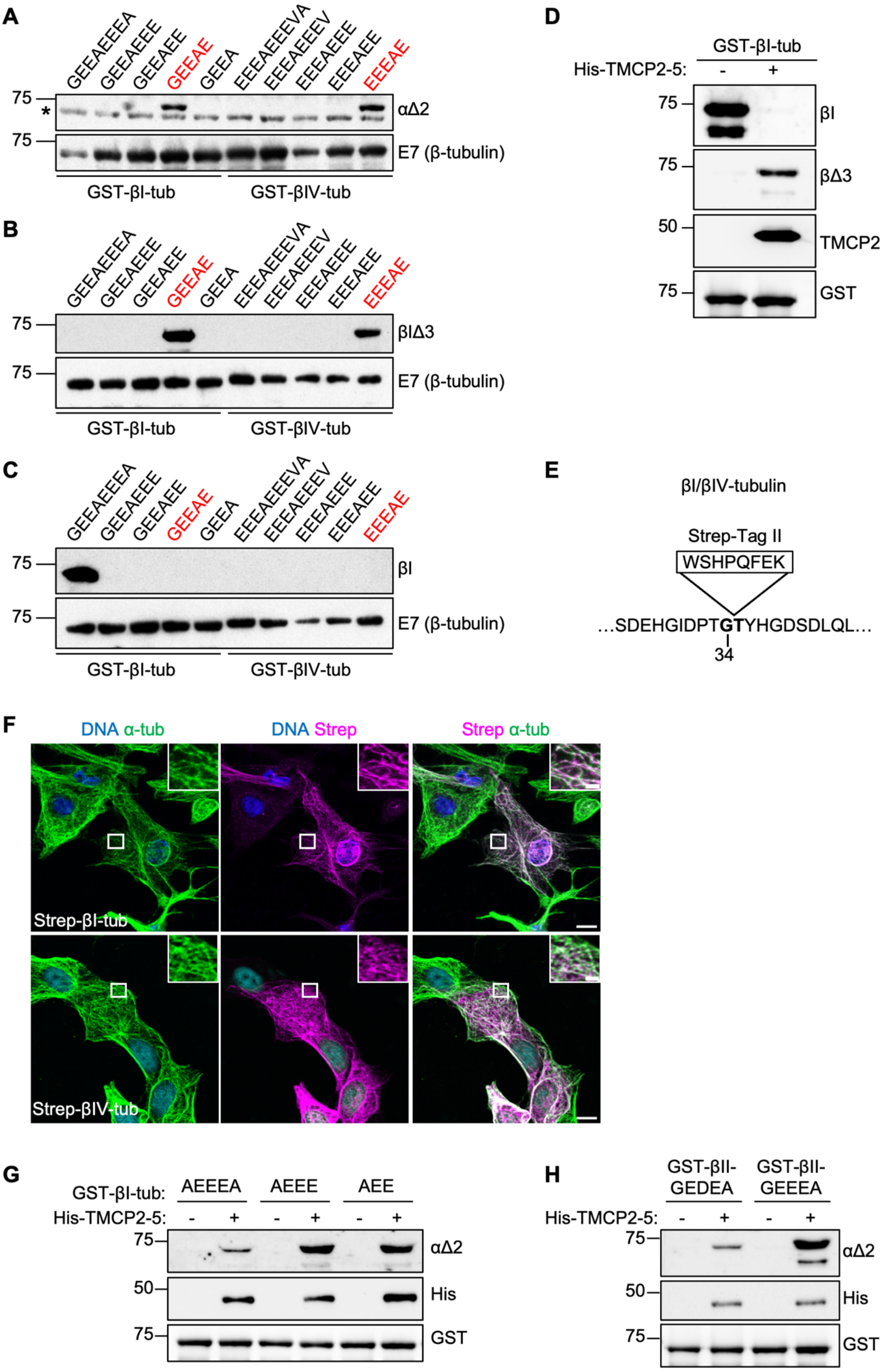

Fig. S6. Characterization of αΔ2, βIΔ3 and βI antibodies as well as TMCP2 activity on various β-tubulin isoforms. (**A-C**) Full-length and C-terminally truncated variants of GST-βI and GST-βIV tubulins were expressed in BL21* bacteria. Immunoblots analysis was performed directly on the crude bacterial extracts. The αΔ2 antibody specifically recognized both β-tubulin tails ending with AE, which corresponds to βIΔ3 and βIVΔ4 tubulins respectively (the epitope is highlighted in red). The asterisk indicates a nonspecific band detected by the αΔ2 antibody. Similarly, the βIΔ3 antibody also specifically recognized βIΔ3 and βIVΔ4 tubulins. The βI antibody only reacted exclusively with full-length βI-tubulin tail. (**D**) Immunoblot analysis of the *in vitro* assay involving TMCP2-5 and GST-βI-tubulin shows a complete disappearance of βI antibody signal with concomitant appearance of βIΔ3 signal in the treated sample. (**E**) Position of the Strep-Tag II sequence inserted into βI- and βIV-tubulins. (**F**) Immunofluorescence images of RPE1 cells transfected with StrepTag-βI or StrepTag-βIV tubulins. The insets show colocalization of strep-tagged tubulins with microtubules indicating a proper incorporation into microtubules. Scale bars: 10 µm, insets: 2 µm. (**G**) Immunoblot analysis of *in vitro* assays involving recombinant His-TMCP2 towards either the wild-type (AEEEA) or truncated variants (AEEE and AEE) of GST-βI-tubulin tail. (**H**) Immunoblot analysis of an *in vitro* assay using recombinant TMPC2-5 and bacterially produced GST-βII-tubulin or its mutated version in which the aspartate has been replaced by glutamate (-GEDEA vs -GEEEA).

Supplementary Figure 7

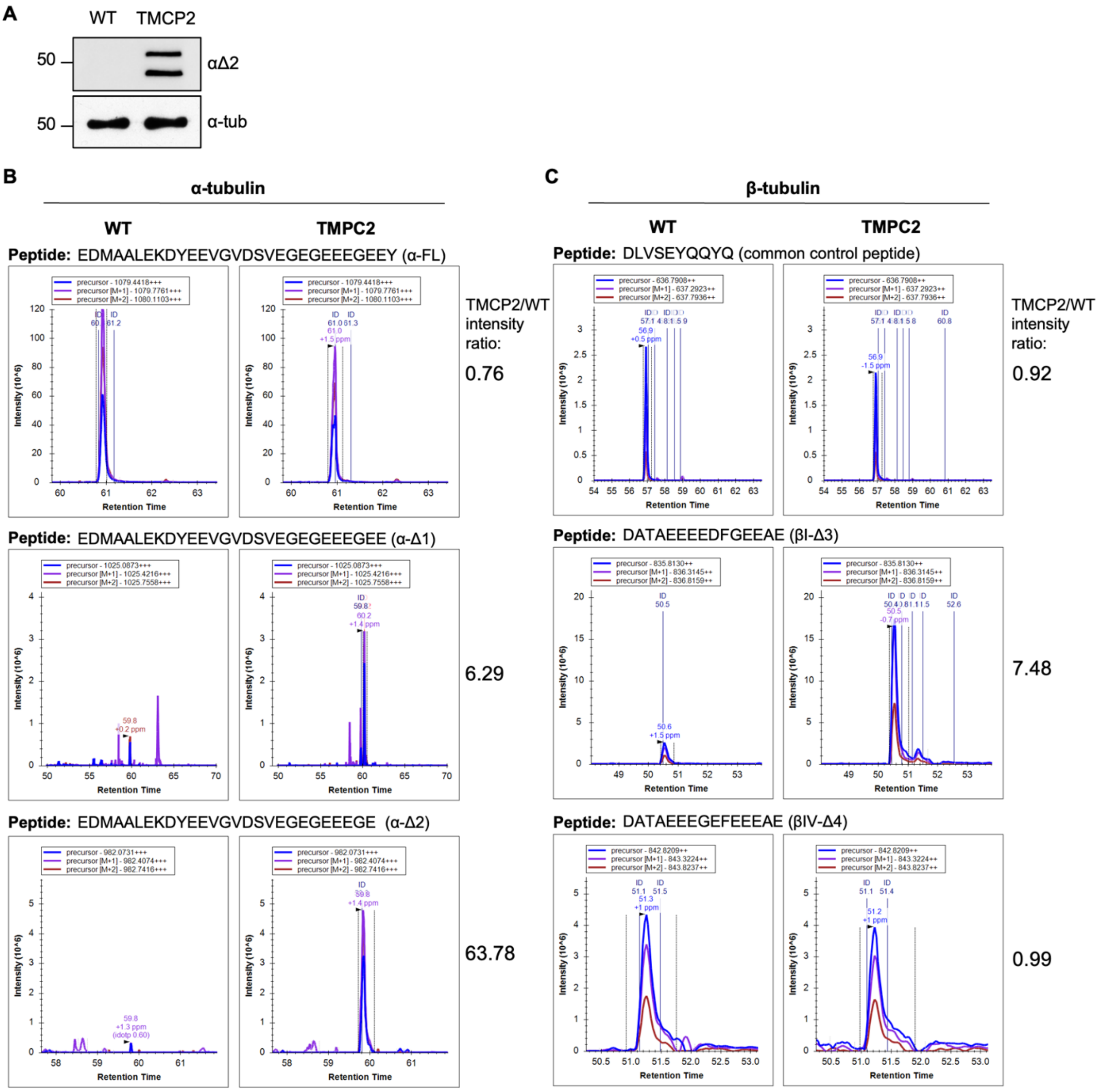

Fig. S7. Mass spectrometry analysis of HEK-2KO-derived tubulin modified by TMCP2. (**A**) Immunoblot of tubulin purified from wild-type or TMCP2-5 expressing HEK-2KO cells. (**B-C**) Mass spectrometry signals were obtained for indicated peptides using Skyline 20.1. The X-axis represents retention time (min) and the Y-axis depicts the intensity (Arbitrary Units). Blue, purple and red chromatograms correspond to major isotope of the precursor. The MSMS spectra that allowed peptide identification are indicated (ID followed by the elution time). The ratios are calculated based on the total area for the selected peaks (dashed grey lines).

Supplementary Figure 8

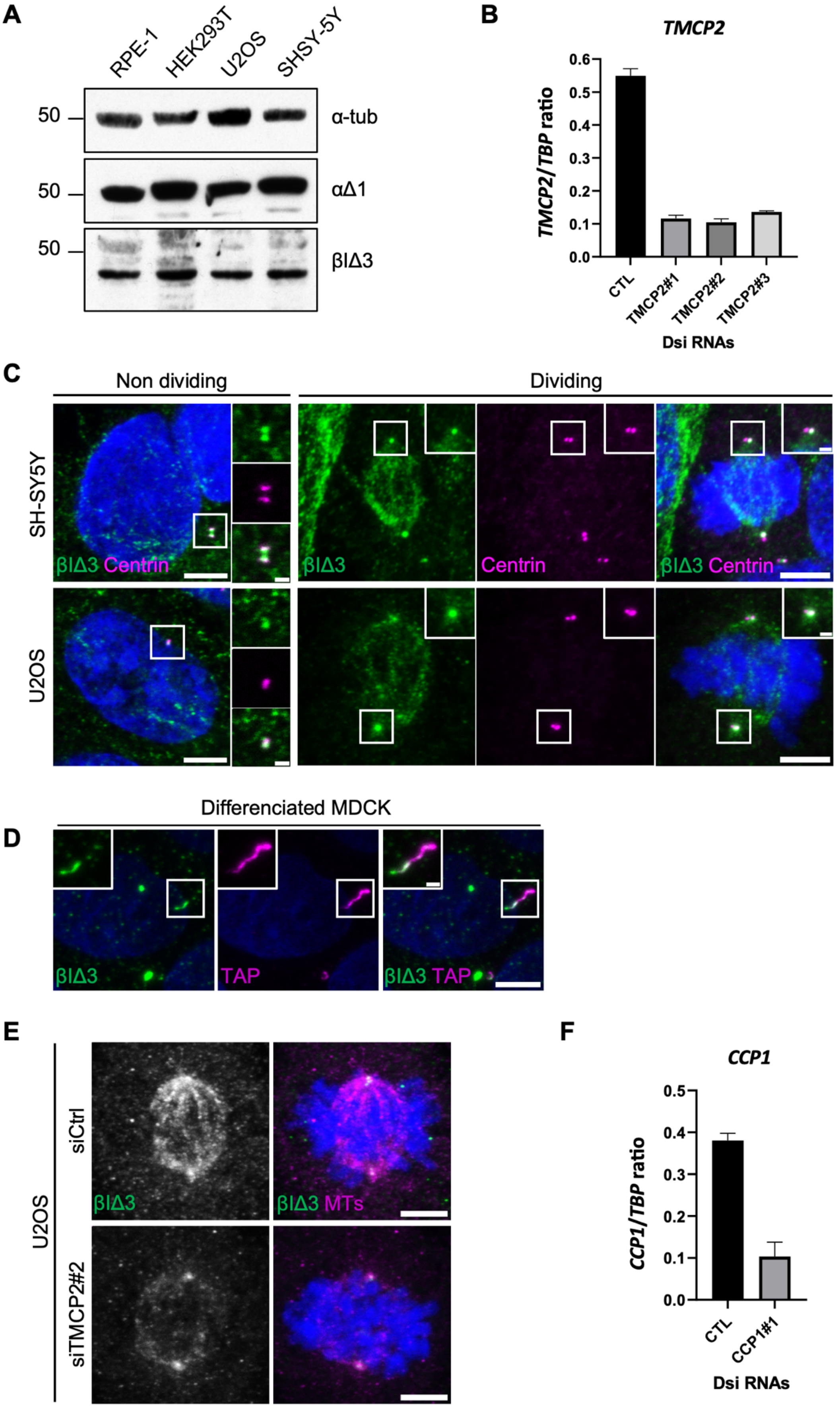

Fig. S8. Detection of endogenous βIΔ3-tubulin in different human cell lines. (**A**) Immunoblot analysis of protein extracts from RPE1, HEK293T, U2OS or SH-SY5Y cells showing the presence of endogenous βIΔ3-tubulin in all cell lines as compared to αΔ1-tubulin and total α-tubulin levels. (**B**) Graphical representation of the knockdown efficiency in SH-SY5Y cells involving three different siRNAs directed against *TMCP2*. Transcripts levels were measured by digital droplet PCR. (**C**) Immunofluorescence analysis of endogenous βIΔ3-tubulin in non-dividing and dividing SH-SY5Y and U2OS cells. As observed in RPE1 cells βIΔ3-tubulin is enriched at centrioles and mitotic spindles (**D**) Immunofluorescence analysis of differentiated MDCK cells showing the accumulation of βIΔ3-tubulin signal at the primary cilia. (**E**) Knock-down of *TMCP2* prevents the accumulation of βIΔ3-tubulin on mitotic spindles of dividing U2OS cells. Scale bars: 5 μm. (**F**) Graphical representation of the knockdown efficiency in SH-SY5Y cells involving an siRNA directed against *CCP1*. Transcripts levels were measured by digital droplet PCR. For all panels scale bars are 5 μm, insets 1 μm.

Supplementary Figure 9

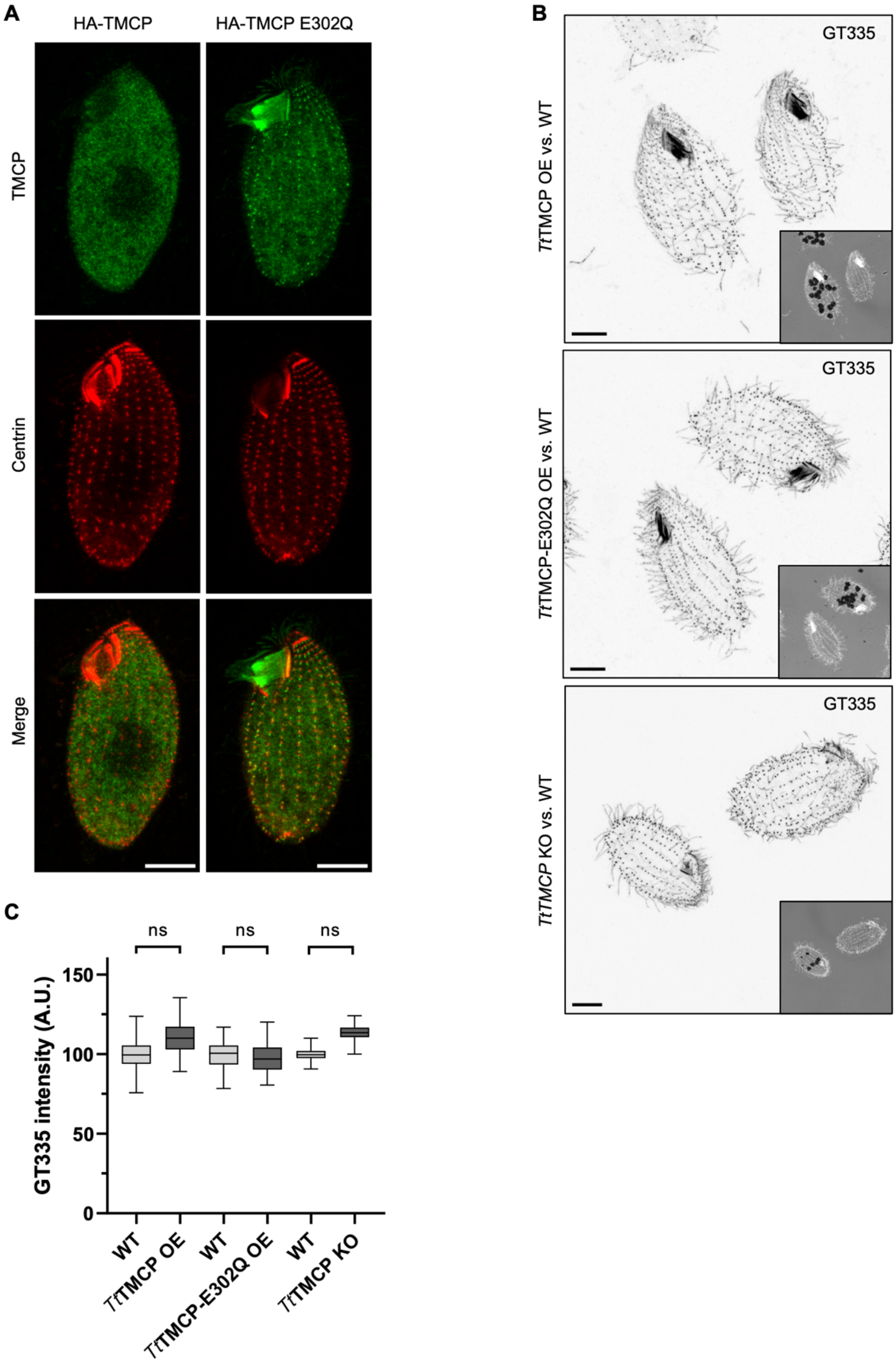

Fig. S9. Characterization of the enzymatic properties of *Tt*TMCP in *Tetrahymena* cells. (**A**) Immunofluorescence analysis of *Tetrahymena* cells showing the localization of HA-*Tt*TMCP or its enzymatically inactive version (E302Q) upon overexpression. Scale bars: 10 µm. (**B**) Immunofluorescence analysis of *Tetrahymena* cells labeled with GT335 antibody (which specifically detects branching point glutamates) upon overexpression (OE) of either GFP-*Tt*TMCP, GFP-*Tt*TMCP-E302Q or cells knockout (KO) for *TtTMCP*. Direct Comparison is made with ink fed wild-type cells on the same coverslip (see the small insets). Scale bars: 10 µm. (**C**) Graphical representation of GT335 signal intensities corresponding to the experiments shown in the panel B (ns=non-significant).

Supplementary Figure 10

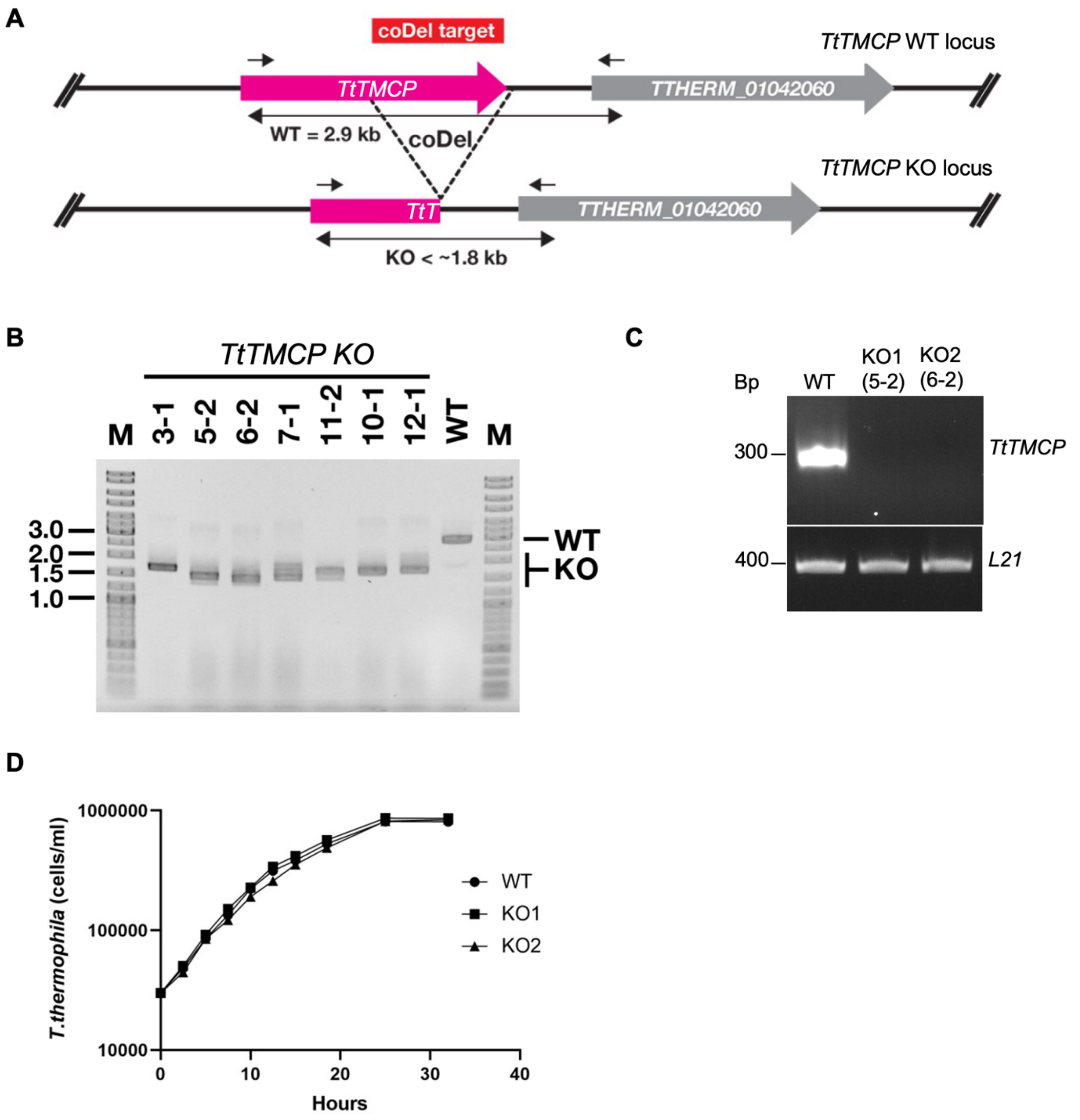

Fig. S10. Generation of *Tetrahymena* strains knockout for TMCP. (**A**) Schematic representation of the wild-type (WT) and the knockout (KO) locus of *TtTMCP*. The target region for co-deletion (coDel) is marked with red box. The positions of the primers used for analyzing locus deletions are shown with arrows. (**B**) Results of genomic PCR detecting deletions at the *TtTMCP* locus. Seven *TtTMCP* knockout cell lines and one wild-type strain were analyzed by direct cell PCR with the primers shown in the top panel. The positions of the PCR products for wild-type (WT) and *TtTMCP* knockout (KO) locus are indicated on right. M = DNA molecular weight marker. (**C**) RT-qPCR analysis of *TtTMCP* RNA levels in wild-type (WT) and two different knockout (KO) strains. The expression of *L21* ribosomal gene was used as a positive control. (**D**) *Tetrahymena* growth curves obtained for wild-type (WT) and two different *TtTMCP* knockout (KO) strains.

Table S1. List of proteins identified by MS, which were enriched in fraction 15 as compared to fraction 11 after gel filtration.
